# Mass Spectrometry–Based Proteomics Analysis of Human Substantia Nigra From Parkinson's Disease Patients Identifies Multiple Pathways Potentially Involved in the Disease

**DOI:** 10.1016/j.mcpro.2022.100452

**Published:** 2022-11-22

**Authors:** Yura Jang, Olga Pletnikova, Juan C. Troncoso, Alexander Y. Pantelyat, Ted M. Dawson, Liana S. Rosenthal, Chan Hyun Na

**Affiliations:** 1 Neuroregeneration and Stem Cell Programs, Institute for Cell Engineering, Johns Hopkins University School of Medicine, Baltimore, Maryland, USA; 2 Department of Neurology, Johns Hopkins University School of Medicine, Baltimore, Maryland, USA; 3 Department of Pathology, Johns Hopkins University School of Medicine, Baltimore, Maryland, USA; 4 Solomon H. Snyder Department of Neuroscience, Johns Hopkins University School of Medicine, Baltimore, Maryland, USA; 5 Department of Pharmacology and Molecular Sciences, Johns Hopkins University School of Medicine, Baltimore, Maryland, USA; 6 Adrienne Helis Malvin Medical Research Foundation, New Orleans, Louisiana, USA; 7 Diana Helis Henry Medical Research Foundation, New Orleans, Louisiana, USA

**Keywords:** Parkinson's disease, substantia nigra, proteomics, human brain tissue, mass spectrometry, mitoribosomes, ACN, acetonitrile, AUC, area under the curve, CAM, cell adhesion molecule, DA, dopamine, FA, formic acid, FDR, false discovery rate, GABA, gamma-aminobutyric acid, HC, healthy control, HCD, higher-energy collisional dissociation, KEGG, Kyoto Encyclopedia of Genes and Genomes, MP, master pool, MRP, mitochondrial ribosomal protein, MS, mass spectrometry, mtDNA, mitochondrial DNA, PD, Parkinson's disease, PMD, postmortem delay, PPI, protein–protein interaction, PSM, peptide-spectrum match, ROC, receiver operating characteristic, RP, ribosomal protein, SAM, significance analysis of microarray, SN, substantia nigra, SNc, SN pars compacta, SNr, SN pars reticulate, TEAB, triethylammonium bicarbonate, TMT, tandem mass tag, WGCNA, weighted gene coexpression network analysis

## Abstract

Parkinson's disease (PD) is the second most prevalent neurodegenerative disorder characterized by the loss of dopaminergic neurons in the substantia nigra (SN) of the brain. Despite decades of studies, the precise pathogenic mechanism of PD is still elusive. An unbiased proteomic analysis of PD patient’s brain allows the identification of critical proteins and molecular pathways that lead to dopamine cell death and α-synuclein deposition and the resulting devastating clinical symptoms. In this study, we conducted an in-depth proteome analysis of human SN tissues from 15 PD patients and 15 healthy control individuals combining Orbitrap mass spectrometry with the isobaric tandem mass tag–based multiplexing technology. We identified 10,040 proteins with 1140 differentially expressed proteins in the SN of PD patients. Pathway analysis showed that the ribosome pathway was the most enriched one, followed by gamma-aminobutyric acidergic synapse, retrograde endocannabinoid signaling, cell adhesion molecules, morphine addiction, Prion disease, and PD pathways. Strikingly, the majority of the proteins enriched in the ribosome pathway were mitochondrial ribosomal proteins (mitoribosomes). The subsequent protein–protein interaction analysis and the weighted gene coexpression network analysis confirmed that the mitoribosome is the most enriched protein cluster. Furthermore, the mitoribosome was also identified in our analysis of a replication set of ten PD and nine healthy control SN tissues. This study provides potential disease pathways involved in PD and paves the way to study further the pathogenic mechanism of PD.

## Highlights

• Proteome analysis of Parkinson’s brains identified >10,000 proteins.
• RNA splicing and complement proteins were upregulated in Parkinson’s brain.
• Mitoribosome proteins were downregulated in Parkinson’s brain.

## Graphical Abstract

**Figure:**
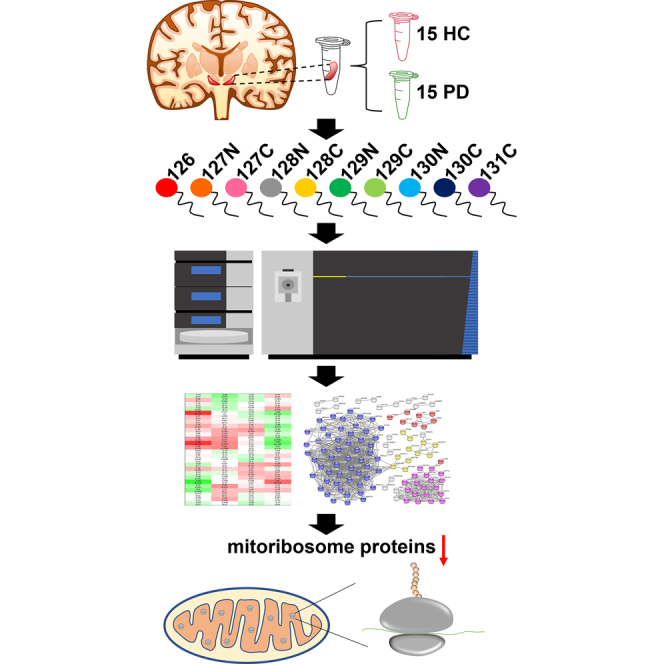

## In Brief

We conducted an in-depth proteome analysis of human substantia nigra tissues from 15 Parkinson’s disease (PD) patients and 15 healthy control individuals to uncover dysregulated pathways in PD. We identified 10,040 proteins with 1140 differentially expressed proteins in the substantia nigra of PD patients, discovering mitoribosome proteins were the most dysregulated proteins, followed by ribosome, RNA splicing, and complement proteins. This study has discovered that mitoribosome dysfunction is potentially involved in the PD pathogenesis process for the first time.

Parkinson's disease (PD) is the second most common neurodegenerative disorder characterized by the loss of dopaminergic neurons in substantia nigra (SN) of the midbrain (1, 2, 3, 4, 5, 6). The primary risk factors for PD are age, environmental influences, and genetic predisposition (7). PD incidence increases with age, with the prevalence of 1% and 4% for people aged over 60 and 80, respectively (8, 9). Exposure to pesticides and heavy metals increases the risk of PD (7). Multiple genes linked to the autosomal dominant form of PD, such as *SNCA*, *LRRK2*, and the autosomal recessive form of PD, such as *PRKN*, *PINK1*, *PARK7* (*DJ1*), and *ATP13A2* (*PARK9*), have been reported (1, 5, 7, 8, 9). SNCA encodes α-synuclein, and one of the typical neuropathologic findings of PD patients is the abnormal deposition of α-synuclein in the cytoplasm of certain neurons (5). The G2019S mutation of *LRRK2* is associated with an impaired lysosomal autophagy system that is critical in the clearance of oligomeric assemblies of α-synuclein (10). In the limited pathologic studies of patients with mutations in Parkin, a ubiquitin E3 ligase, the pattern of dopamine (DA) neuron loss in the SN without the presence of Lewy bodies is shown (8, 9). PINK1 in conjunction with Parkin is highly associated with mitochondria quality control, and the relationship between mitochondrial dysfunction and PD pathogenesis is well known (1). Mutations of *PARK7* are involved in increased oxidative stress, which is linked to the pathogenesis of PD (5). Mutations of *ATP13A2* are associated with the dysregulation of lysosomes and autophagosomes that contribute to PD pathogenesis (5). While these mutations have been noted in genetic forms of PD, the dysfunctional pathways that they lead are also implicated in idiopathic PD (11). Specifically, multiple putative mechanisms are thought to play a role in and include α-synuclein aggregation, mitochondrial dysfunction, abnormal protein clearance, and neuroinflammation among others (1, 5, 12, 13). Aggregated pathologic α-synuclein causes neurotoxicity, and it constitutes the major misfolded proteins found in Lewy bodies (7, 14). Aging, environmental toxins, and genetic predisposition contribute to mitochondrial dysfunction, which is considered a key element in both idiopathic and familial PD (15, 16, 17, 18, 19, 20). Clearance of proteins is an essential cell function to protect cells from proteotoxic stress induced by misfolded and aggregated proteins. Dysfunctional protein clearance is associated with PD pathogenesis. The ubiquitin-proteome system is involved in the clearance of unnecessary proteins in the cell. Thus, the dysregulation of the ubiquitin-proteome system can lead to protein aggregation (21). The autophagy-lysosomal system, which is involved in the degradation of impaired or misfolded proteins through microautophagy and macroautophagy and chaperone-mediated autophagy can also become impaired in PD (22). Neuroinflammation also contributes to pathogenic mechanisms. Levels of inflammatory cytokines that can induce neuronal death are increased in PD (23, 24). Despite these insights into PD pathogenesis, disease-modifying therapy has not been identified, and additional mechanisms remain to be discovered. Along these lines, understanding how the proteome changes in PD patients’ brains may provide novel insights into PD pathogenesis.

Mass spectrometry (MS)–based proteomics technology has been considered the gold standard for proteome analyses and has been applied to study PD (25, 26, 27, 28, 29, 30, 31). Although there have been multiple studies to uncover dysfunctional signaling pathways in SN of PD patients, the number of identified proteins from the studies was still too shallow (<1800 proteins) to uncover key pathways because of the limitation of the used methods and instruments, and/or the number of the samples used was too small (10 or less SN samples) (29, 30, 31). To this end, we conducted an in-depth proteome analysis of human SN tissues from 15 PD patients and 15 healthy control (HC) individuals using Orbitrap MS. In this study, we employed isobaric tandem mass tag (TMT)–based multiplexing for quantification. The validity of the key pathways was independently verified. This is the first report of a large-scale in-depth proteome analysis of human SN in PD *versus* controls and provides a foundation for the elucidation of proteomic changes that contribute to PD pathogenesis.

## Experimental Procedures

### Acquisition of SN Samples

Human SN tissues from 15 PD patients and 15 HC individuals that were used for the acquisition of the discovery data and the human SN tissues from ten PD patients and nine HC individuals that were used for the acquisition of the replication data were acquired from the Brain Resource Center at Johns Hopkins University School of Medicine. The clinical information for the samples is provided in Table 1 and supplemental Table S1. Diagnosis of PD was based on UK Brain Bank clinical criteria and then autopsy confirmation (32, 33). HCs were individuals without clinical or neuropathological evidence of Parkinsonism. All participants agreed to autopsy prior to their death, and their next of kin consented to the autopsy procedure at the time of death. All research was approved by the Johns Hopkins Institutional Review Board. The inclusion criteria for PD are patients with (1) a clinical history of PD with or without dementia; (2) neuropathology changes of Lewy body disease brainstem-predominant, limbic, or neocortical (34); and (3) age older than 50 years, males and females, and any race. The exclusion criteria for PD are patients with any significant neurodegenerative or vascular comorbidity. This study abided by the Declaration of Helsinki principles.

**Table 1:** Information on the SN samples used in the discovery study

No.	Diagnosis	Age at death	Sex	Race	PMD (h)	CERAD
1	PD with dementia, AD definite	64	M	W	21	C
2	PD with dementia, AD probable	82	F	W	5	B
3	PD with dementia, AD probable	80	M	W	13	B
4	PD, no dementia	73	F	W	6	A
5	PD with dementia, LBD neocortical	84	M	W	5	0
6	PD, LBD neocortical, AD	74	M	W	19	C
7	PD, LBD limbic	95	F	W	12	0
8	PD with dementia	76	M	W	19	0
9	PD with dementia	76	M	W	17	0
10	PD with dementia, AD probable	85	F	W	11	B
11	PD, no dementia	86	M	W	22.5	0
12	PD with dementia, AD probable	83	F	W	4	B
13	PD with dementia	60	M	W	15.5	0
14	PD with dementia, AD definite	80	F	W	16	C
15	PD with dementia, AD probable	85	M	W	14	B
16	HC, NFT, & frequent tau neurites in HP (age-associated tau pathology)	76	M	W	25	0
17	HC, moderate Tau+ neurites in HP and ERC	67	M	W	37	0
18	HC, GVD in HP, Tau NFT and neurites in ERC (Braak I)	71	F	B	37	0
19	HC, rare NFT in ERC, no amyloid plaques, old contusions (Braak I)	81	M	W	26	0
20	HC, rare NFTs in ERC and HP, no amyloid plaques (Braak II)	80	F	W	37	0
21	HC, no Tau or amyloid lesions	67	M	W	25	0
22	HC, rare Tau+ neurites in HP, no amyloid plaques	67	M	W	8	0
23	HC, NFT in ERC but not in HP, no amyloid plaques	71	F	W	57	0
24	HC, rare NFT in HP and ERC (Braak II)	66	M	B	25	0
25	HC, NFT in HP and ERC, no amyloid plaques (Braak II)	77	F	W	33	0
26	HC, NFT in ERC but none in HP	80	M	B	21	0
27	HC, mild NFT in HP and ERC, no amyloid plaques (Braak II)	87	F	W	7	0
28	HC, NFT in HP, ERC and ITC, plaques in temporal lobe (Braak III)	90	F	B	22	A
29	HC, no NFT, no amyloid plaques	60	M	W	16	0
30	HC, NFT in ERC and HP, few amyloid plaques in HP	87	F	W	35	A

Abbreviations: AD, Alzheimer’s disease; B, black; CERAD, Consortium to Establish a Registry for Alzheimer’s Disease; ERC, entorhinal cortex; F, female; GVD, granulovacuolar degeneration; HP, hippocampus; ITC, inferior temporal cortex; LBD, Lewy body dementia; M, male; NFT, neurofibrillary tangle; W, white

### Sample Preparation

The SN samples from 15 PD patients and 15 HC individuals were lysed by sonication (Branson Sonifier 250; Branson Ultrasonics) in 8 M urea/50 mM triethylammonium bicarbonate (TEAB). The amount of protein in the samples was quantified using a bicinchoninic acid assay kit (Pierce). To analyze 30 samples using 11-plex TMT method, three batches (sets) of 11-plex TMT experiments were conducted including a reference master pool (MP) in each set. The MP was used for the normalization of the quantification values from the three sets. The MP was prepared by combining an equal amount of protein from all 30 samples. Proteins were reduced and alkylated with 10 mM Tris(2-carboxyethyl) phosphine hydrochloride and 40 mM chloroacetamide at room temperature (22–25 °C) for 1 h. The proteins were then digested with Lys-C (Lysyl endopeptidase MS grade; Fujifilm Wako Pure Chemical Industries Co, Ltd) in a ratio of 1:100 at 37 °C for 3 h. Subsequently, trypsin (sequencing grade modified trypsin; Promega) digestion was conducted by diluting the urea concentration to 2 M by adding the three volumes of 50 mM TEAB followed by adding trypsin in a ratio of 1:50 and incubating at 37 °C overnight (for 15–18 h). The resulting peptides were desalted with C_18_ StageTips (3M Empore; 3M) and labeled with 11-plex TMT reagents according to the manufacturer’s instructions (Thermo Fisher Scientific). The labeling reaction was performed at room temperature for 1 h, followed by quenching with 1/10 volume of 1 M Tris–HCl (pH 8.0). The peptides were pooled and prefractionated by basic pH reversed-phase liquid chromatography into 96 fractions, followed by concatenating into 24 fractions by combining every 24th fraction. The Agilent 1260 offline LC system (Agilent Technologies) was used for basic pH reversed-phase liquid chromatography fractionation, which includes a binary pump, UV detector, an autosampler, and an automatic fraction collector. In brief, the dried samples were reconstituted in solvent A (10 mM TEAB in water, pH 8.5) and loaded onto a column (Agilent 300 Extend-C_18_ column, 5 μm, 4.6 mm × 25 cm; Agilent Technologies). Peptides were resolved using an increasing gradient of solvent B (10 mM TEAB in 90% acetonitrile [ACN], pH 8.5) at a flow rate of 0.3 ml/min. The total run time was 150 min. Subsequently, the concatenated 24 samples were vacuum dried using a SpeedVac (Thermo Fisher Scientific) and then stored at −80 °C until use (24, 35, 36).

The preparation of ten PD and nine HC samples used for the replication was conducted in the same way as described previously, except for the preparation of the MP and the employment of the 10-plex TMT instead of the 11-plex TMT. One HC sample was added to the two sets of 10-plex TMT experiments and used for the normalization of quantification values from the two sets.

### MS

The peptides were analyzed on an Orbitrap Fusion Lumos Tribrid Mass Spectrometer (Thermo Fisher Scientific) coupled with an Ultimate 3000 RSLCnano nanoflow liquid chromatography system (Thermo Fisher Scientific). The peptides from each fraction were reconstituted in 50 μl of 0.5% formic acid (FA), and 30% of reconstituted peptide solution were loaded on a trap column (Acclaim PepMap 100, LC C_18_, 5 μm, 100 μm × 2 cm, nanoViper; Thermo Fisher Scientific) at a flow rate of 8 μl/min. The peptides were resolved at 0.3 μl/min flow rate using an increasing gradient of solvent B (0.1% FA in 95% ACN) on an analytical column (Easy-Spray PepMap RSLC C_18_, 2 μm, 75 μm × 50 cm; Thermo Fisher Scientific), which was fitted with an EASY-Spray ion source that was operated at a voltage of about 2.0 kV. The total run time was 120 min. MS analysis was carried out in data-dependent acquisition mode with a full scan in the mass-to-charge ratio (*m/z*) range of 300 to 1800 in the “Top Speed” mode with 3 s per cycle. MS1 and MS2 were acquired for the precursor ions and the peptide fragmentation ions, respectively. MS1 scans were measured at a resolution of 120,000 at an *m/z* of 200. MS2 scans were acquired by fragmenting precursor ions using the higher-energy collisional dissociation (HCD) method, which was set to 35% of collision energy, and detected at a mass resolution of 50,000 at an *m/z* of 200. Automatic gain control targets were set to one million ions for MS1 and 0.05 million ions for MS2. The maximum ion injection time was set to 50 ms for MS1 and 100 ms for MS2. The precursor isolation window was set to 1.6 *m/z* with 0.4 *m/z* of offset. Dynamic exclusion was set to 30 s, and singly charged ions were rejected. Internal calibration was carried out using the lock mass option (*m/z* 445.12002) from ambient air (24, 35, 36).

The peptides for the replication experiment were analyzed on an LTQ-Orbitrap Elite mass spectrometer (Thermo Fisher Scientific) coupled with an EASY-nano liquid chromatography II system (Thermo Fisher Scientific). The peptides from each fraction were reconstituted in 30 μl of 0.5% FA, and 50% of the reconstituted peptide solution was loaded on the trap column at a flow rate of 10 μl/min. The peptides were resolved at 0.25 μl/min flow rate using an increasing gradient of solvent B (0.1% FA in 95% ACN) on an analytical column (75 μm × 50 cm) that was packed in a house for the LTQ-Orbitrap Elite mass spectrometer. MS analysis was carried out in the data-dependent acquisition with a full scan in the *m/z* range of 300 to 1700 in top N mode setting to eight most intense ions. Full MS scans were measured at a resolution of 120,000 at an *m/z* of 400. MS2 scans were acquired by fragmenting precursor ions using the HCD method and detected at a mass resolution of 30,000 at an *m/z* of 400. Automatic gain control targets were set to one million ions for MS1 and 0.2 million ions for MS2. The maximum ion injection time was set to 100 ms for MS1 and 300 ms for MS2. Dynamic exclusion was set to 60 s, and singly charged ions were rejected. Internal calibration was carried out using the lock mass option (*m/z* 371.101236 and 445.12002) from ambient air.

### Data Analysis

Proteome Discoverer (version 2.2.0.388; Thermo Fisher Scientific) suite was used for quantitation and identification. During MS2 preprocessing, the top ten peaks in each window of 100 Da were selected for database search. The tandem MS data were then searched using SEQUEST HT algorithms against a human UniProt database that includes both Swiss-Prot and TrEMBL (released in May 2018 with 73,112 entries) with common contaminant proteins (115 entries). The search parameters used were as follows: (a) trypsin as a proteolytic enzyme (with up to two missed cleavages); (b) peptide precursor mass error tolerance of 10 ppm; (c) fragment mass error tolerance of 0.02 Da; and (d) carbamidomethylation of cysteine (+57.02146 Da) and TMT tags (+229.16293 Da) on lysine and peptide N termini as fixed modifications; (d) oxidation (+15.99492 Da) of methionine as a variable modification. The minimum peptide length was set to six amino acids, and the minimum number of peptides per protein was set to 1. Peptides and proteins were filtered at a 1% false discovery rate (FDR) at the peptide-spectrum match (PSM) level using a percolator node and at the protein level using the protein FDR validator node, respectively. The protein quantification was performed with the following parameters and methods. The most confident centroid option was used for the integration mode, whereas the reporter ion tolerance was set to 20 ppm. The MS order was set to MS2, and the activation type was set to HCD. Both unique and razor peptides were used for peptide quantification, whereas protein groups were considered for peptide uniqueness. Coisolation threshold was set to 50%. Reporter ion abundance was computed based on signal-to-noise ratios, and the missing intensity values were replaced with the minimum value. The average reporter signal-to-noise threshold was set to 50. The quantification value corrections for isobaric tags and data normalization were disabled. Protein grouping was performed with a strict parsimony principle to generate the final protein groups. All proteins sharing the same set or subset of identified peptides were grouped, whereas protein groups with no unique peptides were filtered out. Proteome Discoverer iterated through all spectra and selected PSM with the highest number of unambiguous and unique peptides, and then final protein groups were generated. The Proteome Discoverer summed all the reporter ion abundances of PSMs for the corresponding proteins in the TMT run (24, 35, 36).

### Experimental Design and Statistical Rationale

The number of SN samples used in this study was 15 PD samples and 15 HC samples for the main experiment and ten PD samples and nine HC samples for the replication experiment. We conducted sample size analysis using the pwr package in R (The R Foundation). When we wanted to detect proteins with 1.5-fold differences between groups, the required minimum sample size was 9.4. When the significance level was 0.0001, power was 0.8, sigma was 0.208, and delta was 0.585 (= log_2_1.5). This sigma value of 0.208 was derived from our in-house TMT proteomics experiments. The significance level of 0.0001 was determined based on our previous studies. When we identified several thousands of proteins, the majority of the proteins with *p* < 0.0001 showed *q* < 0.05. Based on this sample size analysis result, we decided to use 15 samples per group. The statistical analysis was performed with the Perseus software (version 1.6.0.7, Max Planck Institute of Biochemistry). Since we are conducting multiple comparisons, we calculated an FDR by comparing data with and without permutations between groups. For the normalization, the reporter ion intensity values were divided by the MP included in each set followed by dividing by the median values of each protein. The relative abundance values for each sample were *z*-score transformed after log_2_ transformation. We removed proteins with one or more missing values before conducting statistical analysis. To remove batch effects, further normalization was conducted with the ComBat package in R (The R Foundation) (37). Proteins with *q* < 0.05 were considered differentially expressed in PD compared with HC groups. The fold changes between the two groups were calculated by dividing the average abundance values of each protein of PD patients by the ones of HC individuals. According to our normality test using Shapiro–Wilk test in the dplyr package in R, the majority of the proteins showed normal distribution. Thus, *p* values between the two groups were calculated by the Student's two-sample *t* test. The *q* values for the volcano plot were calculated by significance analysis of microarray (SAM) and a permutation-based FDR estimation (38). As an orthogonal method to increase the reliability of the selection for differentially expressed proteins between groups, we also used bootstrap receiver operating characteristic (ROC) curve–based statistical analysis (39, 40, 41, 42). Bootstrap ROC analysis was carried out using the fbroc package in R. The sampling for the bootstrap ROC was conducted with replacement. The area under the curve (AUC) of a bootstrap ROC of two groups in each sampling was computed. Mean and SD values of AUCs from 1000 bootstrap ROC were then calculated (43, 44). The *q* values of bootstrap AUC analysis data were calculated as follows: (1) the mean AUC values for nonpermutated and permuted data were sorted in descending order for proteins with mean AUCs >0.5 and in ascending order for proteins with mean AUCs <0.5; (2) the ratios of the protein numbers for the nonpermuted data to the protein numbers for the permuted data were calculated as lowering the cutoff threshold, and the ratios were used as *q* values.

### Pathway Analysis

The differentially expressed proteins between PD and HC groups in both SAM and bootstrap AUC analyses were used for Kyoto Encyclopedia of Genes and Genomes (KEGG) pathway analysis embedded in DAVID bioinformatics resources (version 6.8, Laboratory of Human Retrovirology and Immunoinformatics) (45, 46). Interactome analysis was carried out by the STRING protein–protein interaction (PPI) databases (version 11) (47, 48). The weighted gene coexpression network analysis (WGCNA) was conducted using the R software package (49, 50).

## Results

### Quantitative Proteome Analysis of SN Samples

**Fig. 1:**
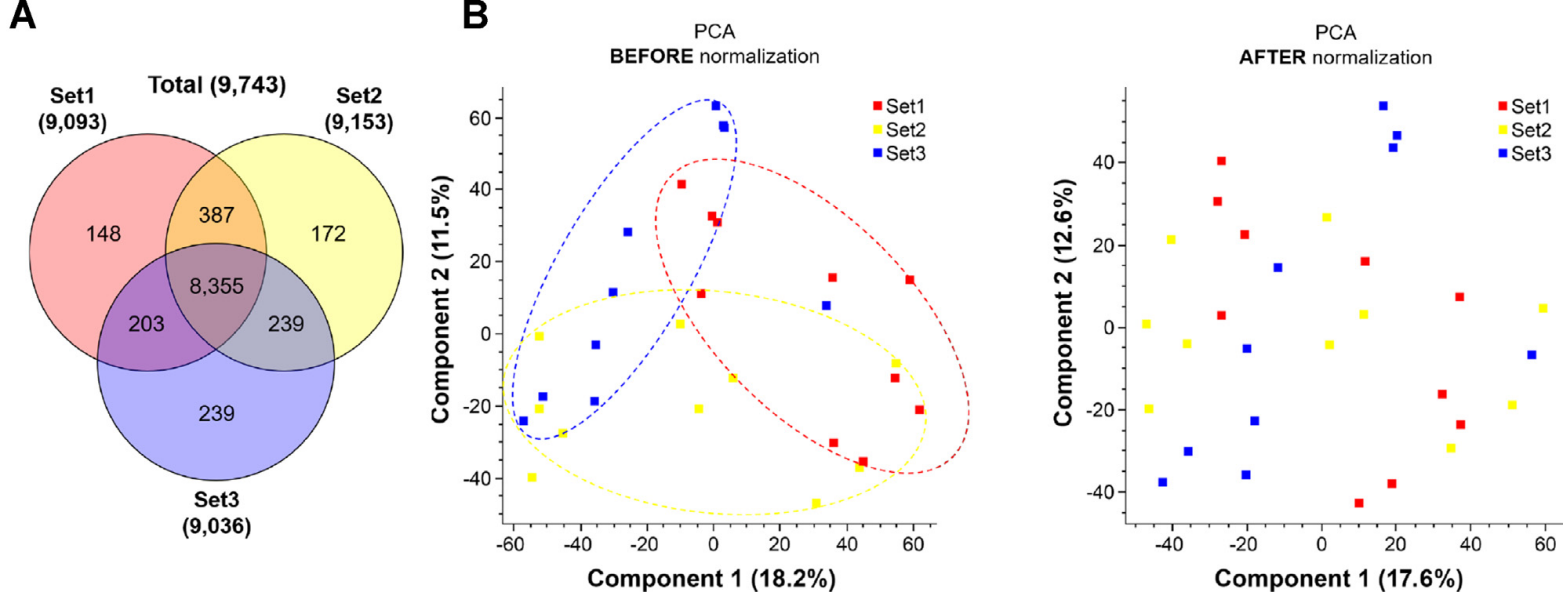
**The number of identified proteins and removal of batch effect by the ComBat package.***A*, the number of the identified proteins in each batch is shown in the Venn diagram. *B*, to minimize batch effects of the three different TMT experiments, they were further normalized using the ComBat package after normalizing each set using MP. Thirty SN samples were shown on a 2D PCA plot to show potential batch effects before (*left panel*) and after (*right panel*) the normalization using the ComBat package. MP, master pool; PCA, principal component analysis; SN, substantia nigra; TMT, tandem mass tag.

To identify differentially expressed proteins in the SN of PD patients, we conducted a quantitative proteome analysis of SN samples from 15 PD patients and 15 HC individuals. For the analysis of 30 SN samples using an 11-plex TMT labeling method, we prepared an MP by pooling a small portion of 30 SN samples. We added the MP to one of the 11 TMT channels in each TMT experimental set for the purposes of normalization (supplemental Fig. S1). The proteins were digested with trypsin and LysC followed by labeling 11-plex TMT reagents. The peptide samples labeled with TMT were prefractionated in 24 fractions with basic pH RPLC and analyzed by LC–MS/MS. In total, 3,167,187 MS/MS spectra were acquired, and 857,332 spectra were assigned to peptides leading to the identification of 134,786 peptides and 9748 proteins. The number of identified proteins from each TMT experimental set and the overlapping proteins among the sets are presented in the Venn diagram (Fig. 1*A*). The numbers of identified proteins from batches 1, 2, and 3 were 9088, 9148, and 9031, respectively (Fig. 1*A* and supplemental Data S1). The number of proteins identified in all batches was 8352. To conduct a statistical analysis of the data acquired from three sets of the TMT experiments, the intensity values of each protein in each set were normalized by the ones of MP. We assessed whether the data from three TMT experiments still retain a batch effect by conducting a principal component analysis. The three sets still showed a residual batch effect (Fig. 1*B*, *left*). To minimize the batch effect, a further normalization was conducted once again using the ComBat package (9). The normalized data by the ComBat package showed more even distribution suggesting that the batch effect was reduced (Fig. 1*B*, *right*).

### Statistical Analysis for the Identification of Differentially Expressed Proteins

**Fig. 2:**
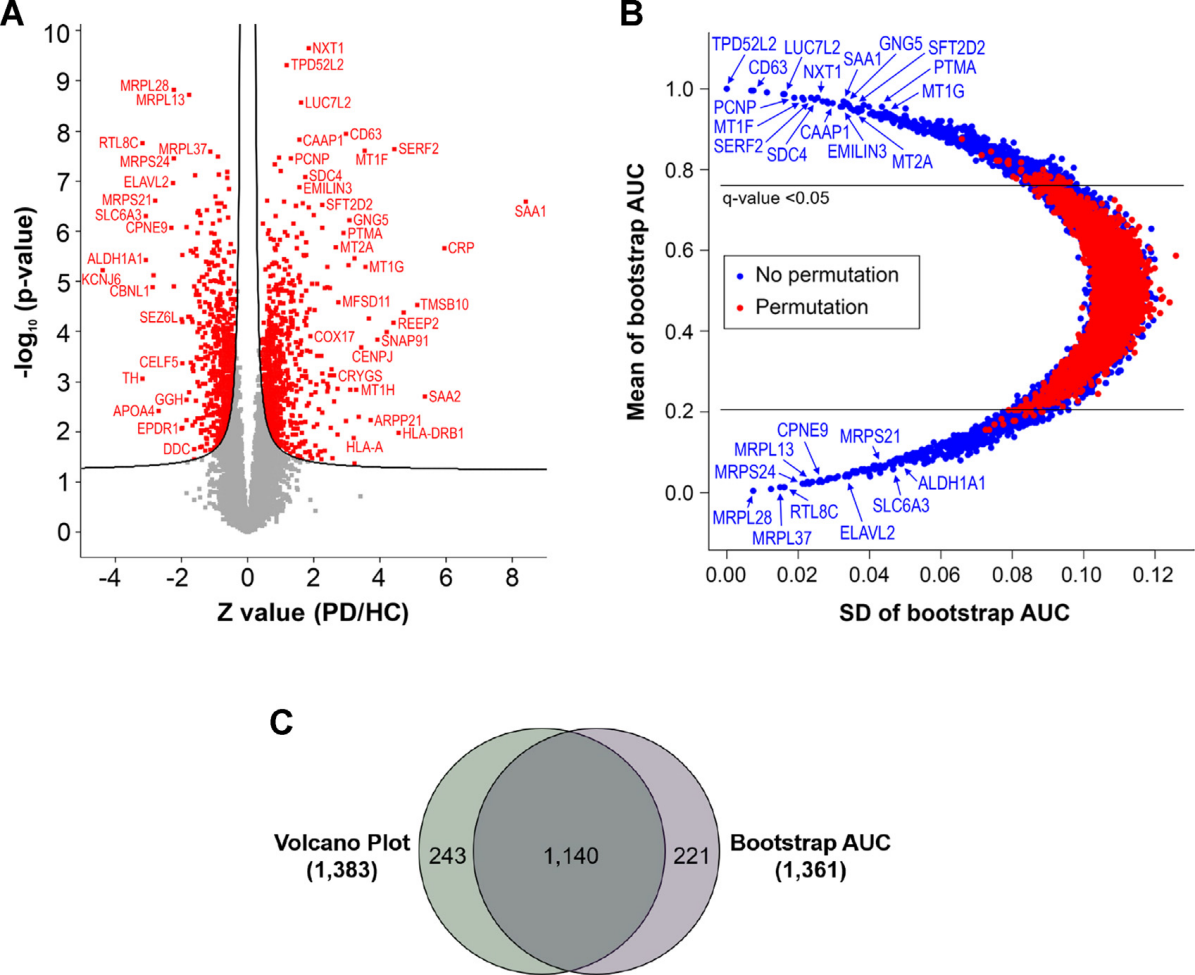
**Volcano plot and bootstrap AUC analysis of the SN proteins identified from PD patients and HC individuals.***A*, the quantified SN proteins from 15 PD patients and 15 HC individuals were plotted on a volcano plot. The *curved line* is the boundary for a *q* value of 0.05. The proteins with *q* < 0.05 are colored in *red font*. The proteins on the *left* and *right sides* of the *q* value line were downregulated and upregulated in PD, respectively. *B*, the quantified SN proteins from 15 PD patients and 15 HC individuals were plotted on a bootstrap AUC plot. The differentially expressed proteins with *q* < 0.05 are shown outside the *horizontal lines*. *C*, the differentially expressed proteins common in the volcano plot and bootstrap AUC analysis are shown in the Venn diagram. AUC, area under the curve; HC, healthy control; PD, Parkinson's disease; SN, substantia nigra.

To identify proteins that are potentially involved in the process of PD pathogenesis, statistical analysis was conducted using two different methods; the SAM-based analysis that uses *p* value and fold change, and the bootstrap ROC–based analysis that uses the AUC and SD of ROCs calculated by random sampling with replacement (supplemental Data S2). The differentially expressed proteins were defined by *q* < 0.05. The number of differential proteins selected by the SAM-based analysis was 1383 (Fig. 2*A* and supplemental Table S2). NXT1, SAA1, TPD52L2, LUC7L2, CD63, CAAP1, SERF2, MT1F, PCNP, SDC4, and so on were the most upregulated proteins, whereas MRPL28, MRPL13, RTL8C, MRPL37, MRPS24, ELAVL2, MRPS21, SLC6A3, CPNE9, and so on were the most downregulated proteins. As expected, ALDH1A1 and TH that are uniquely expressed in dopaminergic neurons also showed approximately eightfold downregulation suggesting dopaminergic neuronal death in the PD patients’ brains. The number of differentially expressed proteins selected by the AUC of the bootstrap ROC was 1361 (Fig. 2*B* and supplemental Table S3). When the list of proteins is sorted by SD value in ascending order, TPD52L2, EIF4B, CD63, MCEE, VAPA, LUC7L2, PCNP, MT1F, NIPBL, SERF2, SDC4, and so on were the most upregulated proteins in PD, whereas MRPL28, hCG_1984214, MRPL37, MRPS9, RTL8C, MRPS24, TIMM23B, MRPL3, MRPL38, LNPEP, and so on were the most downregulated proteins in PD. When the differential proteins from the volcano plot with *q* value of <0.05 were compared with the ones from the bootstrap AUC analysis with *q* value of <0.05, 1140 proteins were common (Fig. 2*C*). We used 1140 proteins that were common between the two analyses for further analysis.

### Gene Set Enrichment Analysis

**Fig. 3:**
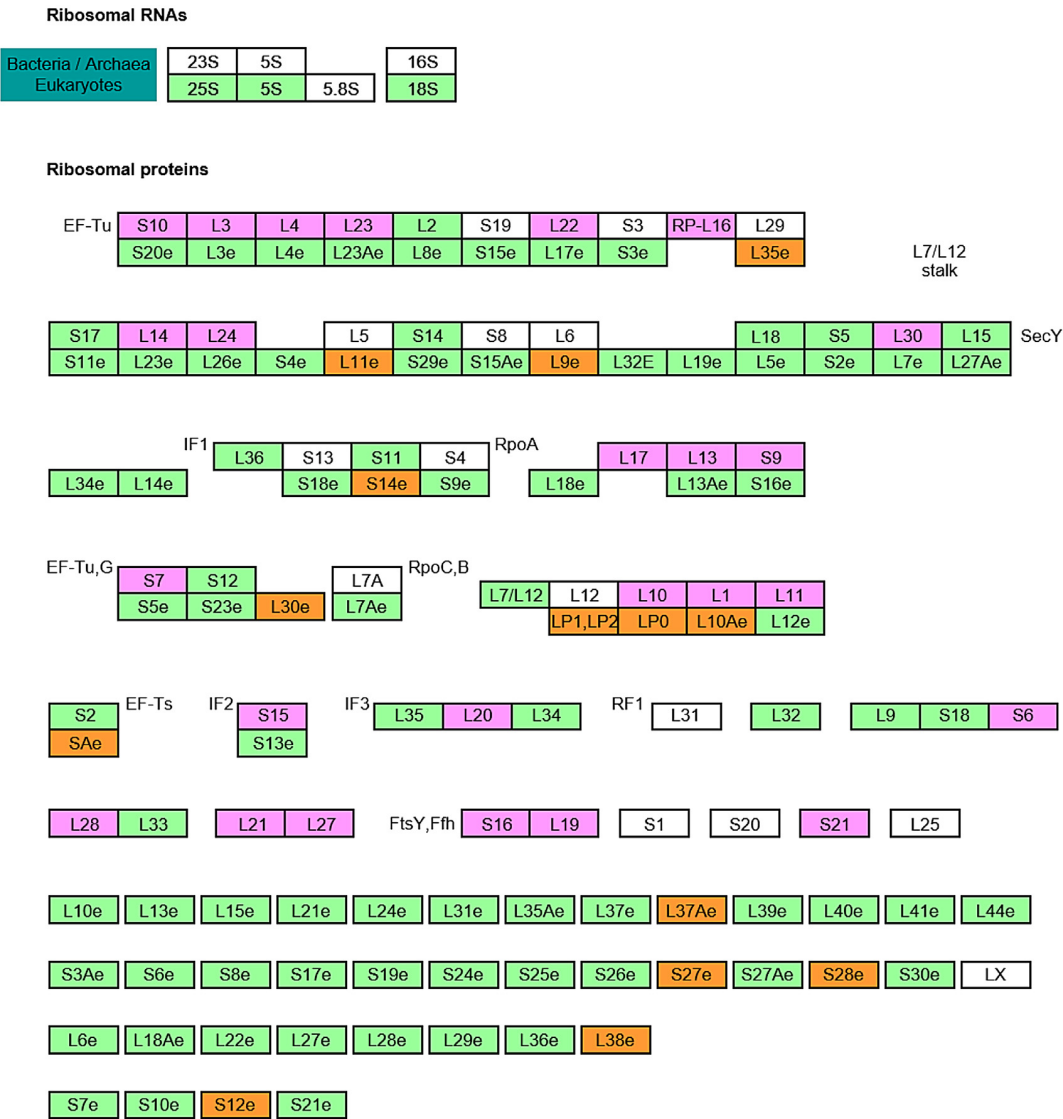
**Ribosome pathway map identified by the gene set enrichment analysis.** The ribosome pathway that was selected as the most enriched one of the differentially expressed proteins in PD using KEGG pathway analysis is displayed here. The ribosomal proteins (RPs) were colored in *orange*, and the mitochondrial ribosomal proteins (MRPs) are colored in *magenta*. KEGG, Kyoto Encyclopedia of Genes and Genomes; PD, Parkinson's disease.

To identify the enriched pathways of the differentially expressed proteins, we conducted gene set enrichment analysis using the KEGG pathway maps. Strikingly, the ribosome pathway was selected as the most enriched pathway, followed by gamma-aminobutyric acid (GABA)ergic synapse, retrograde endocannabinoid signaling, cell adhesion molecules (CAMs), morphine addiction, prion disease, and PD pathways (Table 2 and supplemental Table S4). The ribosome pathway was enriched with 42 proteins with *p* value of 1.4 × 10^−16^ (Fig. 3). Of 42 proteins, 17 and 25 proteins were ribosomal proteins (RPs) and mitochondrial ribosomal proteins (MRPs), respectively. Among the 17 RPs, two proteins were upregulated and 15 proteins were downregulated in PD (supplemental Table S5). Among 25 MRPs, all of them were downregulated in PD (supplemental Table S6). The GABAergic synapse pathway was enriched with 18 proteins with a *p* value of 6.2 × 10^−5^. Eight of 18 proteins were GABA receptor proteins, and three of 18 were guanine nucleotide–binding proteins. The retrograde endocannabinoid signaling pathway was enriched with 18 proteins with a *p* value of 5.5 × 10^−4^. Five of 18 proteins were GABA receptor proteins, and four of 18 were guanine nucleotide–binding proteins. The CAM pathway was enriched with 22 proteins with *p* value of 7.8 × 10^−4^, and three of them were integrin proteins. The morphine addiction pathway was enriched with 16 proteins with *p* value of 1.4 × 10^−3^. Seven of 16 proteins were GABA receptor proteins, and three of 18 were guanine nucleotide–binding proteins. The prion disease pathway was enriched with nine proteins with *p* value of 1.9 × 10^−3^, and three of them were complement proteins. The PD pathway was enriched with 20 proteins with a *p* value of 4.4 × 10^−3^, and seven of them were NADH dehydrogenase subcomplex proteins. Interestingly, three pathways, GABAergic synapse, retrograde endocannabinoid signaling, and morphine addiction, share GABA receptors and guanine nucleotide–binding proteins, and these shared proteins contribute to the enriched pathways. These results suggest that the main protein clusters formed by differentially expressed proteins in SN of the PD brain are MRPs, RPs, GABA receptors, and NADH dehydrogenase subcomplex proteins.

**Table 2:** Enriched pathways of the differentially expressed proteins

Term	Count/PH	Percent	*p*
Ribosome	42/136	30.9	1.40E-16
GABAergic synapse	18/85	21.2	6.20E-05
Retrograde endocannabinoid signaling	18/101	17.8	5.50E-04
CAMs	22/142	15.5	7.80E-04
Morphine addiction	16/91	17.6	1.40E-03
Prion diseases	9/34	26.5	1.90E-03
PD	20/142	14.1	4.40E-03

Abbreviation: PH, the total number of proteins in the pathway.

### Interactome Analysis

**Fig. 4:**
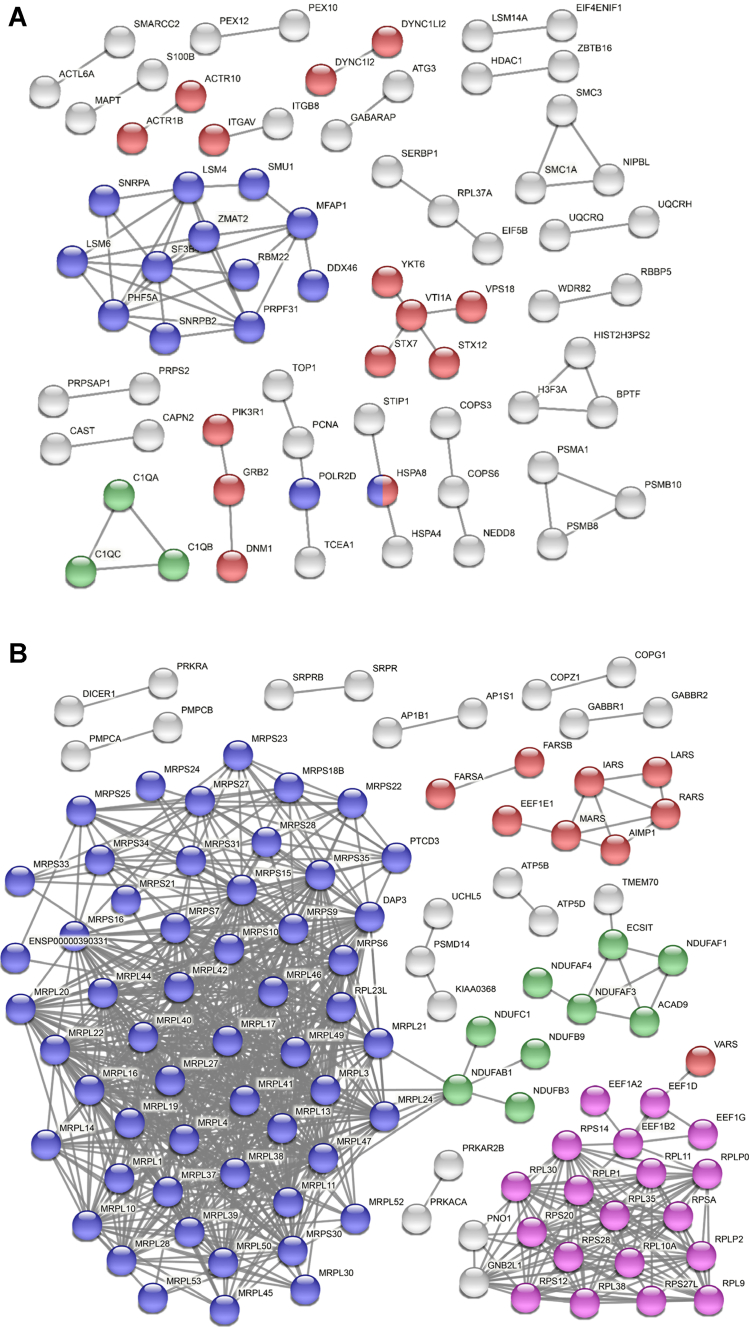
**STRING PPI analysis of the differentially expressed proteins in PD.***A*, STRING PPI analysis was conducted to estimate the connectivity of the upregulated proteins. The network contains 634 nodes with 70 edges. The experiment alone was used as an active interaction source with the highest confidence threshold of 0.9 (average node degree: 0.221, average local clustering coefficient: 0.0946, and PPI enrichment *p* value: 0.00013). The *blue*, *red*, and *green nodes* denote RNA splicing (GO: 0008380), vesicle-mediated transport (GO: 0016192), and complement cascade pathways (HSA-166658), respectively. The *gray nodes* belong to other pathways. *B*, STRING PPI analysis was conducted to estimate the connectivity of the downregulated proteins. The network contains 502 nodes with 821 edges. The experiment alone was used as an active interaction source with the highest confidence threshold of 0.9 (average node degree: 3.27, average local clustering coefficient: 0.189, and PPI enrichment *p* value: <1.0E-16). The *blue*, *pink*, *green*, and *red nodes* denote mitochondrial gene expression (GO: 0140053), eukaryotic translation elongation (HSA-156842), respiratory electron transport (HSA-611105), and tRNA aminoacylation for protein translation (GO: 0006418), respectively. The *gray nodes* belong to other pathways. GO, Gene Ontology; PD, Parkinson's disease; PPI, protein–protein interaction.

Although we have identified a few enriched pathways for the differentially expressed proteins in the SN of the PD patients, we reasoned that an orthogonal analysis would enable us to narrow down key pathways. For this, we conducted an interactome analysis with the upregulated and downregulated proteins to unravel key functional modules using the STRING functional protein association network (48, 51). For the upregulated proteins, RNA splicing–related proteins formed the most connected cluster followed by vesicle-mediated transport and complement cascade pathways (Fig. 4*A*). While activated immune response is a well-known factor in PD pathogenesis, only three proteins formed a small cluster. Thus, we investigated how many differentially expressed immune response–related proteins were identified. We could identify nine immune response–related proteins differentially expressed. Interestingly, all the differentially expressed inflammation-related proteins, complement proteins (C1Q, C9, C1B, C1C, C4B, C4A, CFHR1, and C1S), and interferon-gamma receptor 1 were increased, clearly showing that the activated complement proteins are one of the potential main causative factors of PD (supplemental Data S2). For the downregulated proteins, the MRPs formed the most connected cluster, followed by RPs (Fig. 4*B*). Since MRPs and RPs formed large clusters only for the downregulated proteins, we investigated how many of the MRPs and RPs were downregulated among all the identified proteins. Interestingly, all the MRPs and the majority of RPs were downregulated in the SN from PD patients (Tables 3 and 4). The human genome has 85 RPs and 78 MRPs in the UniProt knowledgebase (52). In this study, we identified 81 RPs and 70 MRPs. While 19 (23%) of 81 RPs were dysregulated, 51 (73%) of 70 MRPs were dysregulated in the SN of the PD patients. Other than RPs, respiratory electron transport proteins and tRNA aminoacylation–related proteins formed clusters too. These results suggest that mitochondrial ribosomal functions were more severely compromised in the SN of the PD patients’ brains, followed by the functions of RPs, spliceosome proteins, respiratory complex proteins of mitochondria, vesicle-mediated transport proteins, and complement cascade proteins.

**Table 3:** List of differentially expressed RPs

Protein name	Protein symbol	*p*	*q*	*z*-score (PD/HC)
60S ribosomal protein L36a-like	RPL36AL	5.02E-06	0	2.42
60S ribosomal protein L37a	RPL37A	0.006372	0.009278	1.75
Ribosomal protein S6 kinase alpha-5	RPS6KA5	0.004994	0.031981	0.65
Ribosomal protein S6 kinase alpha-3	RPS6KA3	1.09E-05	0.017386	−0.41
60S ribosomal protein L30	RPL30	0.002938	0.046668	−0.46
60S ribosomal protein L11	RPL11	0.000192	0.009427	−0.61
60S ribosomal protein L38	RPL38	0.000162	0.007457	−0.65
60S acidic ribosomal protein P0	RPLP0	0.000124	0.004742	−0.72
40S ribosomal protein S27-like	RPS27L	0.002075	0.01566	−0.76
40S ribosomal protein S20	RPS20	0.000877	0.0098	−0.76
60S acidic ribosomal protein P2	RPLP2	2.59E-05	0.001787	−0.78
40S ribosomal protein S28	RPS28	2.22E-05	0.001374	−0.82
40S ribosomal protein S14	RPS14	0.000389	0.005355	−0.83
60S ribosomal protein L9	RPL9	2.32E-06	0.000464	−0.84
40S ribosomal protein SA	RPSA	1.60E-06	0.000325	−0.86
40S ribosomal protein S12	RPS12	4.69E-06	0.00046	−0.91
60S ribosomal protein L10a	RPL10A	1.67E-06	0.000233	−1.00
60S acidic ribosomal protein P1	RPLP1	1.12E-06	0.000222	−1.00
60S ribosomal protein L35	RPL35	0.001919	0.00586	−1.24

**Table 4:** List of differentially expressed mitochondrial RPs

Protein name	Protein symbol	*p*	*q*	*z*-score (PD/HC)
28S ribosomal protein S21, mitochondrial	MRPS21	2.51E-07	0	−2.81
28S ribosomal protein S24, mitochondrial	MRPS24	3.57E-08	0	−2.24
39S ribosomal protein L28, mitochondrial	MRPL28	1.51E-09	0	−2.24
39S ribosomal protein L23, mitochondrial	MRPL23	5.13E-05	0.000202	−1.81
39S ribosomal protein L13, mitochondrial	MRPL13	1.91E-09	0	−1.78
39S ribosomal protein L21, mitochondrial	MRPL21	2.46E-06	0	−1.73
28S ribosomal protein S34, mitochondrial	MRPS34	2.61E-06	0	−1.63
28S ribosomal protein S9, mitochondrial	MRPS9	7.78E-08	0	−1.58
39S ribosomal protein L41, mitochondrial	MRPL41	2.26E-06	0	−1.57
28S ribosomal protein S16, mitochondrial	MRPS16	1.49E-05	0.000211	−1.55
28S ribosomal protein S7, mitochondrial	MRPS7	3.14E-06	0.000114	−1.55
39S ribosomal protein L3, mitochondrial	MRPL3	4.22E-07	0	−1.53
28S ribosomal protein S25, mitochondrial	MRPS25	1.09E-06	0	−1.52
39S ribosomal protein L24, mitochondrial	MRPL24	9.94E-06	0.000225	−1.47
28S ribosomal protein S10, mitochondrial	MRPS10	6.81E-07	0	−1.43
28S ribosomal protein S31, mitochondrial	MRPS31	0.000464	0.001912	−1.30
28S ribosomal protein S35, mitochondrial	MRPS35	0.000333	0.001547	−1.28
28S ribosomal protein S22, mitochondrial	MRPS22	0.001102	0.003783	−1.28
39S ribosomal protein L42, mitochondrial	MRPL42	4.10E-06	0.000215	−1.25
39S ribosomal protein L47, mitochondrial	MRPL47	6.97E-05	0.000741	−1.20
39S ribosomal protein L49, mitochondrial	MRPL49	2.82E-05	0.000442	−1.17
39S ribosomal protein L19, mitochondrial	MRPL19	5.71E-06	0.00022	−1.15
39S ribosomal protein L37, mitochondrial	MRPL37	2.60E-08	0	−1.14
39S ribosomal protein L53, mitochondrial	MRPL53	9.83E-06	0.000291	−1.12
39S ribosomal protein L44, mitochondrial	MRPL44	1.60E-05	0.000383	−1.09
39S ribosomal protein L1, mitochondrial	MRPL1	0.000131	0.001516	−1.04
28S ribosomal protein S27, mitochondrial	MRPS27	0.000629	0.004009	−1.03
28S ribosomal protein S18b, mitochondrial	MRPS18B	2.53E-06	0.000215	−1.03
28S ribosomal protein S15, mitochondrial	MRPS15	0.000321	0.002953	−1.00
28S ribosomal protein S23, mitochondrial	MRPS23	0.000929	0.00569	−0.99
39S ribosomal protein L16, mitochondrial	MRPL16	2.06E-07	0.0002	−0.98
28S ribosomal protein S6, mitochondrial	MRPS6	0.000179	0.00238	−0.97
39S ribosomal protein L50, mitochondrial	MRPL50	0.000187	0.002615	−0.95
39S ribosomal protein L30, mitochondrial	MRPL30	0.0006	0.0048	−0.95
39S ribosomal protein L45, mitochondrial	MRPL45	0.000201	0.002754	−0.94
28S ribosomal protein S33, mitochondrial	MRPS33	0.00118	0.007917	−0.91
39S ribosomal protein L20, mitochondrial	MRPL20	2.30E-05	0.001	−0.90
39S ribosomal protein L10, mitochondrial	MRPL10	0.000118	0.002372	−0.90
39S ribosomal protein L38, mitochondrial	MRPL38	3.08E-06	0.000337	−0.90
28S ribosomal protein S28, mitochondrial	MRPS28	3.69E-05	0.001225	−0.89
39S ribosomal protein L46, mitochondrial	MRPL46	0.001204	0.008648	−0.87
39S ribosomal protein L40, mitochondrial	MRPL40	4.80E-05	0.001552	−0.87
39S ribosomal protein S30, mitochondrial	MRPS30	2.48E-05	0.001151	−0.87
39S ribosomal protein L17, mitochondrial	MRPL17	1.01E-05	0.000979	−0.84
39S ribosomal protein L52, mitochondrial	MRPL52	0.000731	0.007355	−0.83
39S ribosomal protein L27, mitochondrial	MRPL27	0.000305	0.004996	−0.81
39S ribosomal protein L11, mitochondrial	MRPL11	0.000853	0.009026	−0.79
39S ribosomal protein L22, mitochondrial	MRPL22	6.40E-06	0.00107	−0.77
39S ribosomal protein L14, mitochondrial	MRPL14	0.00237	0.016919	−0.75
39S ribosomal protein L39, mitochondrial	MRPL39	3.86E-05	0.002897	−0.73
39S ribosomal protein L4, mitochondrial	MRPL4	0.003948	0.037657	−0.55

### Coexpression Analysis Using WGCNA

**Fig. 5:**
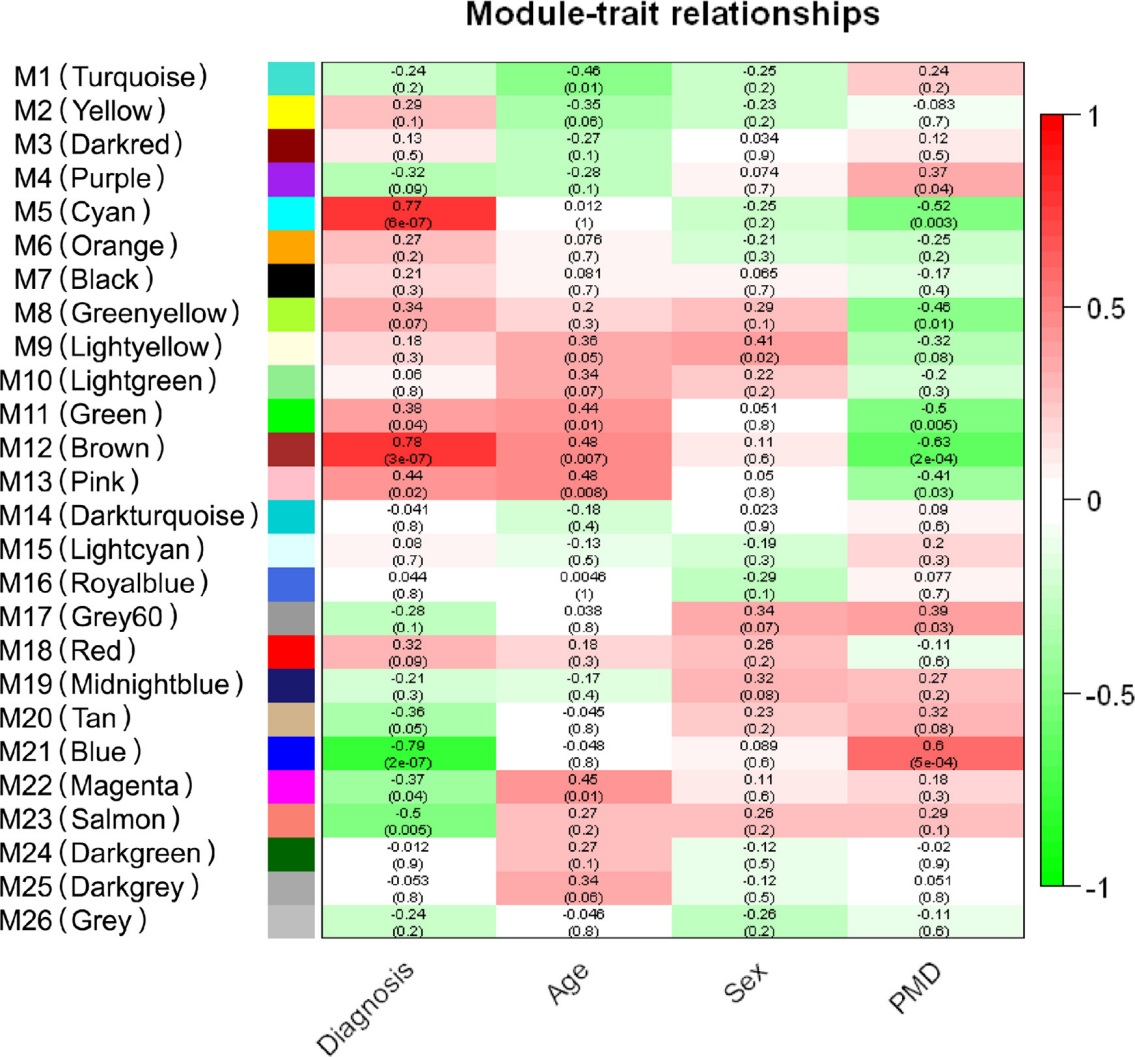
**The module–trait relationships of the WGCNA of SN proteome data.** The module–trait relationships of WGCNA of the SN proteome data were presented in the form of a heatmap. The Pearson correlations between 26 protein cluster modules and four traits composed of diagnosis, age, sex, and PMD were calculated and colored on a scale of 1 (positive correlation) to −1 (negative correlation). A protein cluster module was generated by collecting proteins with similar expression patterns across the samples. The correlation values are shown at the *top* of each box, and the *p* values are shown on the *bottom* of each box inside the parenthesis. PMD, postmortem delay; SN, substantia nigra; WGCNA, weighted gene coexpression network analysis.

The gene set enrichment and interactome analyses of the differential proteins in the SN of the PD patients’ brains suggested that mitochondrial ribosome could be the most affected pathway in the PD brains. However, we still could not rule out the possibility that this pathway could be identified by other traits of the samples than the PD pathology. To address this, we conducted an unbiased coexpression analysis using WGCNA, which clusters proteins with similar patterns and calculates correlations of the 26 protein cluster modules with various traits of the samples, such as diagnosis, age, sex, and postmortem delay (PMD) (Table 1 and supplemental Data S3) (50). The WGCNA results showed that the M5 (*cyan*), M11 (*green*), M12 (*brown*), and M13 (*pink*) modules showed a positive correlation (*p* < 0.05) with PD implying that the proteins in the clusters have a pattern of increased expression level in the PD samples. On the other hand, M21 (*blue*), M22 (*magenta*), and M23 (*salmon*) modules showed a negative correlation (*p* < 0.05) with PD implying that the proteins in the cluster have a pattern of decreased expression level in the PD samples (Fig. 5 and supplemental Fig. S2). Because the gene set enrichment and interactome analyses showed that MRPs were decreased in PD, we postulated that the MRPs would be clustered in the modules that had the pattern of decreased expression level in the PD samples. For this reason, we conducted a KEGG pathway analysis with the proteins in the M21, M22, and M23 modules to identify the module that has enriched MRPs. The M21 module showed the most significant enrichment with MRPs (supplemental Fig. S3*A* and supplemental Table S7, *top*). In addition, the M23 module also showed the most significant enrichment with RPs (supplemental Fig. S3*B* and supplemental Table S7, *bottom*). Although the M21 module showed almost no correlation with age and sex, it showed a mild positive correlation with PMD. These results suggest that there is the possibility that PMD could affect the identification of mitochondrial ribosomes as an enriched pathway in the PD samples. To rule out this possibility, we conducted statistical analysis by classifying samples into two groups based on PMD. The PMD was divided into low and high based on its median value, resulting in the regrouping of three participants. The statistical analysis results showed no differential proteins, suggesting that the downregulated MRPs are not correlated to PMD but to PD (supplemental Fig. S4).

### An Independent Replication Experiment of the Pathways Discovered in the Main Experiment

Gene set enrichment and interactome analyses showed that the ribosome pathway, especially MRPs, is a key protein cluster linked to PD pathology. However, we still cannot exclude the possibility that the dysregulated ribosome pathway was a feature unique to the SN samples that we used in the main experiment. Thus, we reasoned that if we could observe similar results from an independent experiment using a different cohort of SN samples, we could have higher confidence in the identified pathways. For this, we analyzed the proteome data of SN from ten PD patients and nine HC individuals that was acquired before the main experiment was conducted by an independent researcher using a different mass spectrometer. Statistical and data analysis were performed in the same way as the main experiment (supplemental Figs. S5 and S6 and supplemental Data S4). The gene set enrichment analysis showed that the ribosome pathway was the most enriched pathway, as was observed in the main experiment (supplemental Table S8, supplemental Fig. S7, and supplemental Data S5). The interactome analysis also showed that the MRPs and RPs were the most connected clusters, as observed in the main experiment (supplemental Fig. S8). We identified 76 RPs and 51 MRPs, and 44 (58%) of 76 RPs and 36 (71%) of 51 MRPs were dysregulated in SN of PD patients. This replication experiment suggests that the ribosome pathway discovered in the main experiment is linked to PD pathology with high confidence.

## Discussion

In this study, we conducted MS-based proteome analysis of human SN brain tissue samples from 15 PD patients and 15 HC individuals using the TMT labeling method. This is the first in-depth proteome analysis of the human SN region from PD patients and HC individuals in which we identified ∼10,000 proteins. In this study, we conducted two different statistical analyses, the SAM-based one and the bootstrap ROC–based one, to find differentially expressed proteins between the two groups. The SAM-based statistical analysis is the most widely used in the proteomics field. However, while conducting SAM-based statistical analysis, variable *q* value cutoff lines can be generated depending on the S_0_ values that users set. When the S_0_ value is 0, the *q* value cutoff line is solely affected by *p* values. As the S_0_ value increases, more weight is given to the fold change than the *p* value in determining the *q* value cutoff line. Therefore, the proteins in the proximity of the *q* value cutoff line are subjected to be included or excluded depending on the S_0_ value that users set. To minimize this ambiguity, we added another layer of statistical analysis by employing the bootstrap ROC. Since bootstrap analysis uses resampling approaches, it outperforms Student's *t* statistics in finding true-positive and true-negative proteins (53). Thus, the two different statistical analyses employed in this study would be helpful in sifting true-positive differentially expressed proteins with reduced ambiguity. Gene set enrichment analysis using differentially expressed proteins in PD showed that ribosome, GABAergic synapse, retrograde endocannabinoid signaling, CAM, morphine addiction, prion diseases, and PD pathways were the most enriched ones, suggesting that they could be potentially involved in the PD pathogenesis. Strikingly, the majority of the RPs enriched in the gene set enrichment analysis were mitoribosomes. The subsequent STRING PPI analysis and WGCNA also showed that mitoribosomes formed the largest highly connected cluster. In addition, more than 50% of the proteins enriched in the PD pathway are mitochondria-related proteins. These results indicate that many mitochondria-related proteins are dysregulated in the SN of PD patients, consistent with many previous reports of abnormal mitochondrial function in PD (18, 19, 31, 54, 55).

Previously, van Dijk *et al.* (56) performed the proteomic analysis with human locus coeruleus brain tissues from six PD patients and six HC individuals identifying 2495 proteins with 87 differential proteins. They discovered that the main affected pathways were mitochondrial dysfunction, oxidative stress, protein misfolding, cytoskeleton dysregulation, and inflammation. Lachén-Montes *et al.* (57) performed proteome analysis with human olfactory bulb tissues from 12 PD patients and eight HC individuals, quantifying 1629 proteins with 268 differentially expressed proteins. They discovered modulation in ERK1/2, MKK3/6, and PDK1–PKC signaling axis. Basso *et al.* (29) performed proteome analysis with human SN brain tissues from four PD patients and four HC individuals identifying 44 proteins with nine proteins with abundance change. Werner *et al.* (30) performed proteome analysis with human SN tissues from five PD patients and five HC individuals identifying 38 proteins with 16 differentially expressed proteins. They discovered alterations of GSH-related proteins as well as alterations of proteins involved in retinoid metabolism. Licker *et al.* (31) performed proteome analysis with human SN from three PD patients and three HC individuals employing a TMT-based LC–MS/MS analysis identifying 1795 proteins with 204 differentially expressed proteins. They discovered that the most altered pathways were mitochondrial dysfunction, oxidative stress, or cytoskeleton impairment. Choi *et al.* (58, 59) and Gómez and Ferrer (60) also performed proteome analysis with human cortex brain tissues from PD patients. Choi *et al.* (58, 59) reported altered expression of ubiquitin carboxyl-terminal hydrolase L1 and oxidative damage of DJ1 in the PD brain. Gómez and Ferrer (60) reported oxidative damage of aldolase A, enolase 1, and glyceraldehyde dehydrogenase. Consistent with our study, these proteomics studies of PD using human brain tissues suggested that the affected pathways in the PD brains were mitochondrial dysfunction. In addition, these other proteomic studies indicated that oxidative stress, protein misfolding, cytoskeleton impairment, and inflammation play a role in the pathogenesis of PD. Although mitochondrial dysfunction is well known in PD, little is known about the involvement of mitoribosomes. Billingsley *et al.* (61) reported that MRPS34, a mitoribosome, could be a PD risk gene. Since mitoribosomes are involved in the translation of mitochondrial proteins encoded by mitochondrial DNA (mtDNA), the downregulated mitoribosome would affect the translation of mitochondrial proteins encoded by mtDNA (mtDNA-encoded proteins). Our study showed that four of five mtDNA-encoded proteins show a trend of downregulation in SN of PD, but those mtDNA-encoded proteins did not show statistically significant differences. There are multiple explanations for why mtDNA-encoded protein levels did not change while mitoribosome proteins were downregulated. The first possible explanation is that mtDNA-encoded proteins were downregulated in neuronal cells but upregulated in other cell types. When we analyze the mixture of proteins from multiple cell types, the outcome of the summed protein abundance often misleads the interpretation of the results. The second possible explanation is that the mtDNA-encoded proteins have longer protein turnover, and they were less affected by the downregulation of mitoribosomes. Thus, cell type–specific proteome analysis and protein turnover study on mitochondrial proteins would provide a clue on why mtDNA-encoded proteins were not downregulated.

GABAergic synapse, retrograde endocannabinoid signaling, and morphine addiction pathways that were enriched with GABA receptor proteins suggested that GABA-related pathways were also potentially compromised in the SN of PD patients. The direct relevance between PD pathogenesis and the GABAergic system is unknown, but their potential indirect relevance has already been reported by several research groups (62, 63). For example, although SN does not have GABAergic neurons, the SN pars reticulata (SNr) region has receptors for GABAergic projection exons (64). It is known that DA depletion induced by dopaminergic neuronal death in SN pars compacta (SNc) of PD patients affects GABAergic transmission in basal ganglia and this, in turn, possibly affected the expression of GABAergic receptors in SNr (65). Therefore, dysregulation of GABA receptor proteins in the SN of PD could be considered a consequence of dopaminergic neuronal death. At a glance, it would be considered that the downregulation of GABAergic receptors will lead to the upregulation of glutamatergic neurons. We identified 20 glutamate receptor proteins in this study. Interestingly, three proteins with *q* < 0.05 (SAM analysis) were downregulated, and most of the remaining proteins also showed a trend of downregulation although their *q* values >0.05 (supplemental Table S9). According to the basal ganglia neural circuit, SNr receives GABAergic transmission from the caudate/putamen and SNr sends GABAergic transmission to the thalamus. On the other hand, both SNc and SNr receive glutamatergic transmissions from the subthalamic nucleus. Subsequently, SNc sends a dopaminergic transmission to caudate/putamen, and SNr sends GABAergic transmission to the thalamus (66, 67). Therefore, the downregulated GABAergic receptors in SNr will result in reduced GABAergic transmission from SNr to the thalamus, not affecting glutamatergic transmission in SN. So, the downregulated glutamatergic receptors in SN discovered in this study can be explained by dopaminergic neuronal death in SNc because the dopaminergic neuronal death will result in the loss of glutamatergic receptors on dopaminergic neurons. Another possible explanation of the downregulated glutamatergic receptors is that it was the consequence of downscaling of the glutamatergic receptors caused by constitutive glutamatergic stimulation. It is already known that the consistent stimulation of DA neurons by glutamatergic stimulation from the subthalamic nucleus is involved in PD pathogenesis (68).

Further study to understand their correlation is required. The PD pathway was enriched with 20 differentially expressed proteins. TH and SLC6A3, which are dopaminergic neuron-specific proteins, showed downregulation (69). SLC18A2, a transmembrane protein that transports monoamines, also showed downregulation. When SLC18A2 function is inhibited, DA cannot be released into the synapse *via* a typical release mechanism (70). The downregulation of TH, SLC6A3, and SLC18A2 can be explained by dopaminergic neuronal death in SN. On the other hand, GPR37, which is a putative substrate of Parkin, was increased. This protein is known to be linked to juvenile PD, and misfolded GPR37 has been found in Lewy bodies. Elderly GPR37 knockout mice displayed deficits in motor performance, and properly folded GPR37 can have a neuroprotective effect (71). UBE2L3 is an E2 ubiquitin–conjugating enzyme that plays a role in Parkin-mediated mitochondrial elimination (72). COX6B1, COX7B, NDUFA1, NDUFA4L2, NDUFAB1, NDUFB2, NDUFB3, NDUFB9, NDUFC1, UQCRH, and UQCRQ are mitochondrial proteins (73, 74, 75). Thus, the dysregulation of UBE2L3, COX6B1, COX7B, NDUFA1, NDUFA4L2, NDUFAB1, NDUFB2, NDUFB3, NDUFB9, NDUFC1, UQCRH, and UQCRQ is potentially linked to mitochondrial dysfunction too. In addition to the proteins that were manifested in the gene set enrichment analysis, NXT1, SAA1, TPD52L2, LUC7L2, CD63, CAAP1, SERF2, MT1F, PCNP, and so on were significantly upregulated, and RTL8C, ELAVL2, CPNE9, ALDH1A1, KCNJ6, and so on were significantly downregulated in PD. A strong increase of a metallothionein protein, MT1F, in the astrocytes in PD SN was previously reported consistent with our findings (76). ALDH1A1 is involved in the catabolism of reactive DA metabolites in dopaminergic neurons (77), and the reduction of ALDH1A1 in PD SN reflects the loss of dopaminergic neuronal functions. However, little is known about the relevance of the rest of the proteins to PD.

In addition to the pathways revealed by the gene set enrichment analysis, the STRING PPI analysis exhibited highly clustered nodes that were not revealed by the gene set enrichment analysis, such as RNA splicing–related proteins, vesicle-mediated transport proteins, complement cascade–related proteins, and tRNA aminoacylation–related proteins. The implication of aberrant alternative splicing of PD-related proteins in the PD pathogenesis has been reported; alternative splicing of *SNCA* can accelerate or decelerate the aggregation of α-synuclein, several pathogenic mutations affect *LRRK2* alternative splicing, and alternative spliced *PARK2* (*Parkin*) variants are implicated in juvenile Parkinsonism (78, 79, 80). The dysregulation of the vesicle-mediated transport pathway is also well known to be involved in PD pathogenesis (81, 82). For example, *VPS35*, which is one of the known PD-related genes, encodes the protein that transports endosomal cargoes to vesicles and tubes, and the mutation on *VPS35* results in the dysregulation of the vesicle transports (83). The complement cascade proteins also have been reported to be involved in PD pathogenesis (24, 84, 85, 86). Ma *et al.* (24) reported that the complement and coagulation cascade has been dysregulated in two representative PD mouse models. Loeffler *et al.* (86) reported activation of the complement pathway in the SN of PD patients. Gregersen *et al.* (85) reported α-synuclein-mediated activation of the classical complement pathway in α-synuclein-expressing cellular model. However, little is known about the involvement of tRNA aminoacylation–related proteins. It seems that the cluster formation of tRNA aminoacylation–related proteins could be caused by the downregulation of RPs.

To analyze 30 samples, we conducted three batches of TMT experiments in this study. Although the three batches of 11-plex TMT-based data were normalized by the reference sample, an obvious batch effect was observed, and further normalization by the ComBat package minimized it. This result suggests that simple normalization by a common reference sample is not enough to remove the batch effect when multiple batches of TMT experiments are conducted. In this study, we discovered that multiple dysregulated pathways occurred in PD patients’ brains, and especially the mitochondrial pathway was the most dysregulated one. We cannot exclude the possibility that these pathways are only observable during the terminal stage since the tissue samples used in this study were from postmortem brains at the terminal stage of PD. Recently, we reported the α-synuclein gut-to-brain propagation mouse model that best recapitulates the Braak hypothesis (87). The SN proteome change of the mouse model over the disease progression would potentially provide a clue when the mitoribosome dysfunction appears. Furthermore, we should deconvolute which cell types manifest this mitoribosome dysfunction through cell type–specific proteome analysis. Despite these limitations, this study has discovered that mitoribosome dysfunction is potentially involved in the PD pathogenesis process for the first time, and this study paves the way to future studies investigating mechanisms of PD pathogenesis.

## Data and Software Availability

All MS data and search results have been deposited to the ProteomeXchange Consortium *via* the PRIDE partner repository with the dataset identifier PXD037684 and project name “Mass spectrometry-based proteomics analysis of human substantia nigra from Parkinson's disease patients identifies multiple pathways potentially involved in the disease.”

## Supplemental data

This article contains supplemental data.

## Conflict of interest

The authors declare no competing interests.
