# Supplementary material for: Mass Spectrometry–Based Proteomics Analysis of Human Substantia Nigra From Parkinson's Disease Patients Identifies Multiple Pathways Potentially Involved in the Disease
Source: Mol Cell Proteomics. 2022 Nov 22;22(1):100452. doi: 10.1016/j.mcpro.2022.100452 (PMC9792365; doi:10.1016/j.mcpro.2022.100452)

Supplemental Figure S1

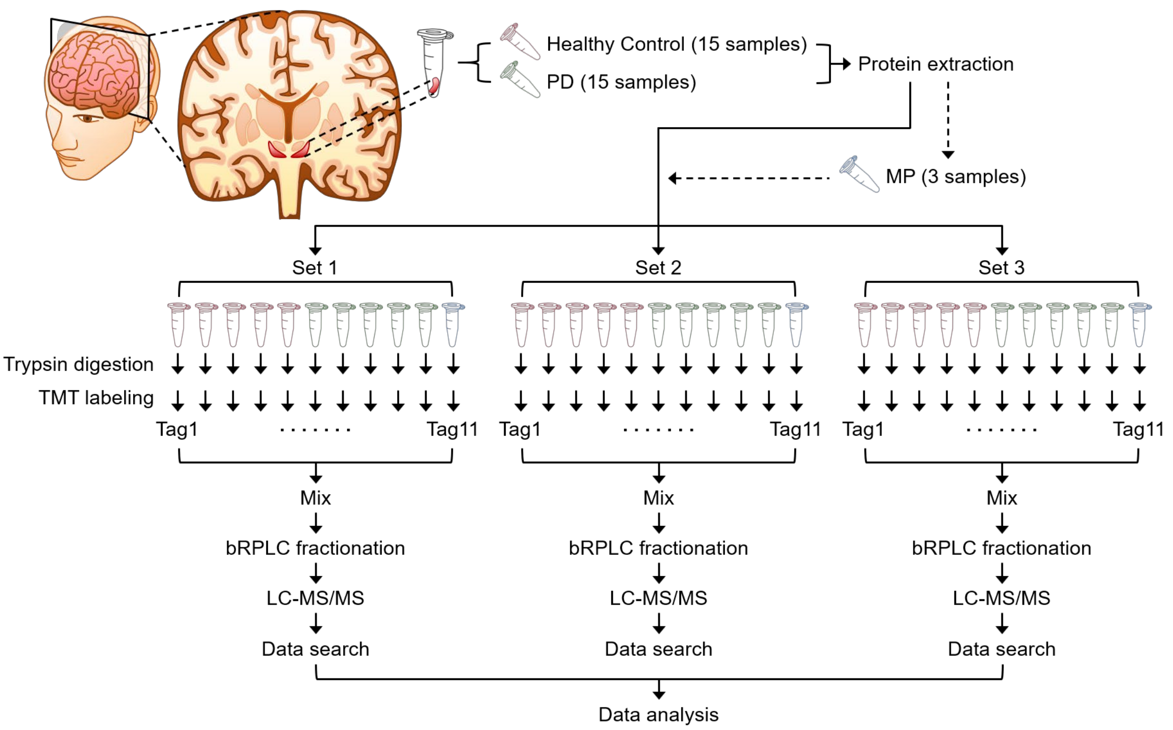

Supplemental Figure S2

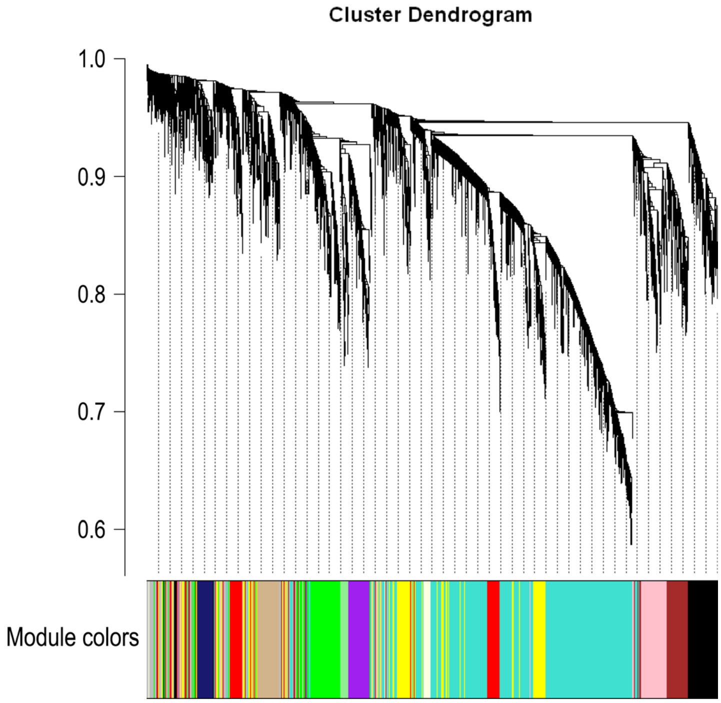

Supplemental Figure S3

(A)

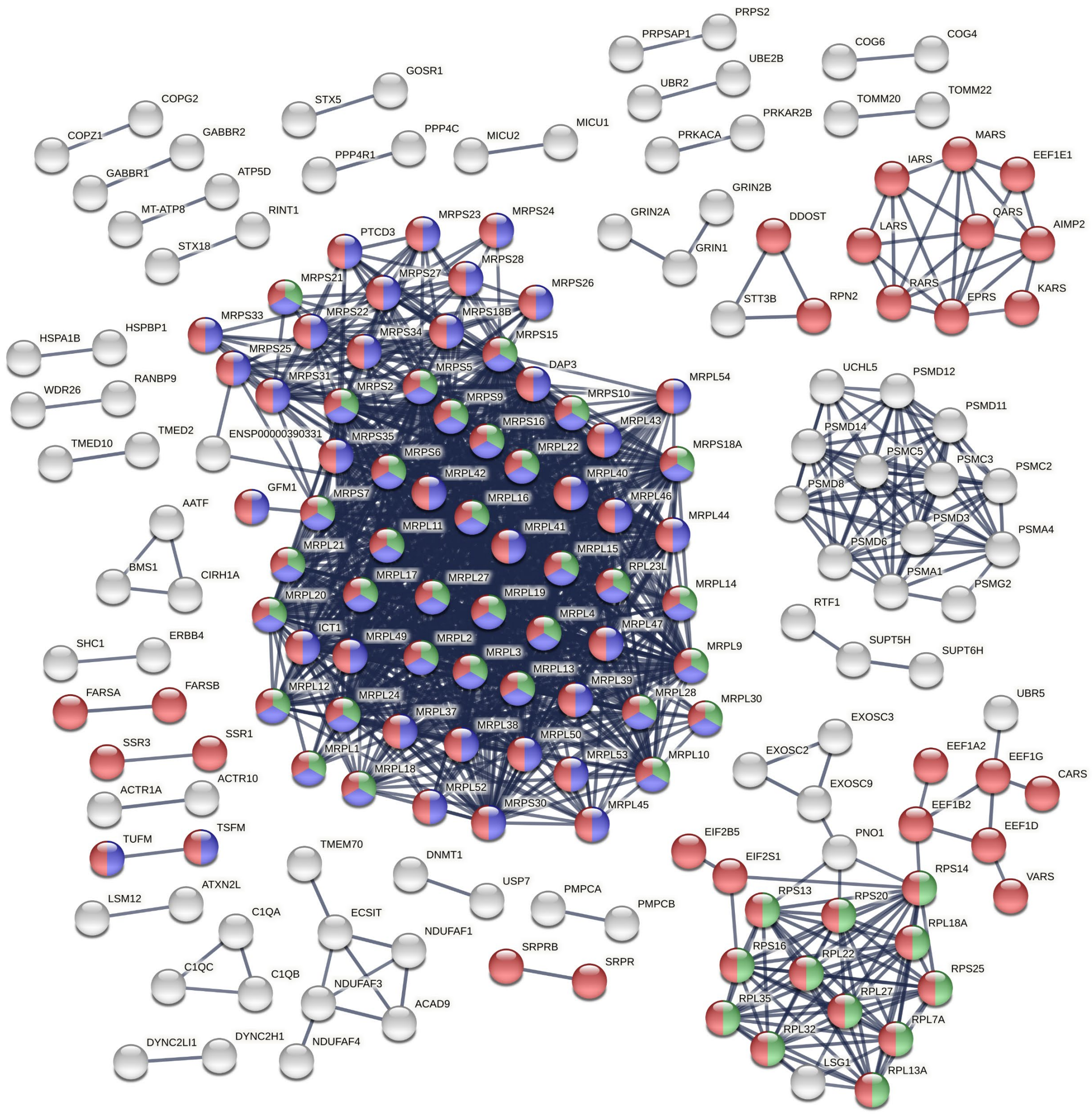

(B)

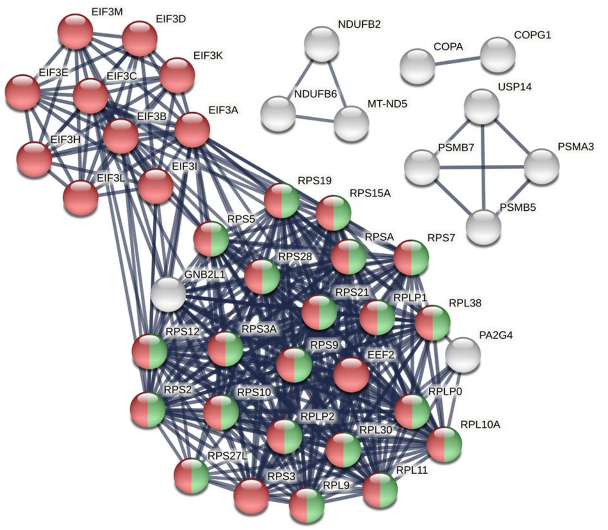

Supplemental Figure S4

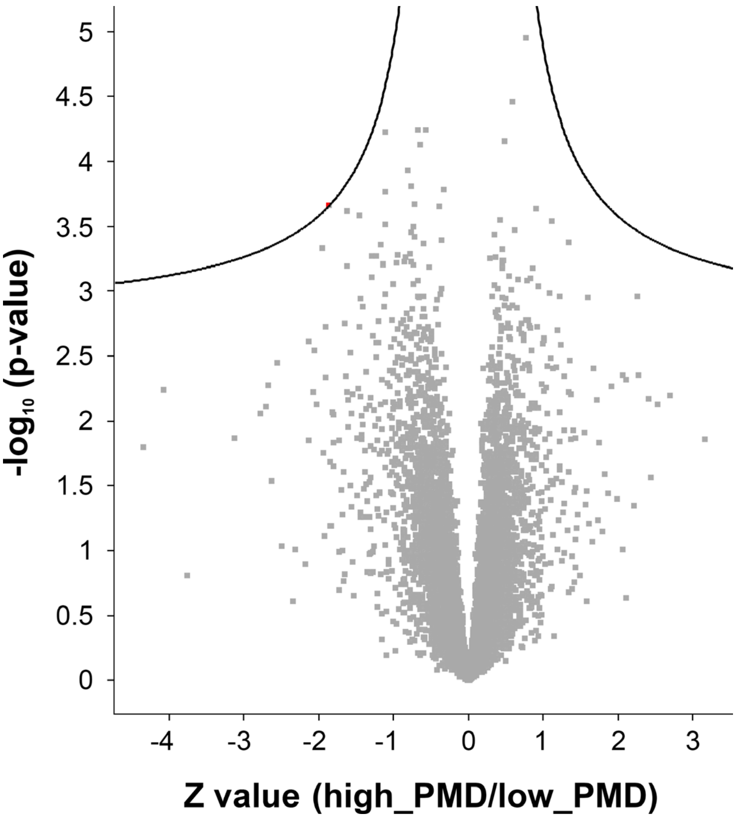

Supplemental Figure S5

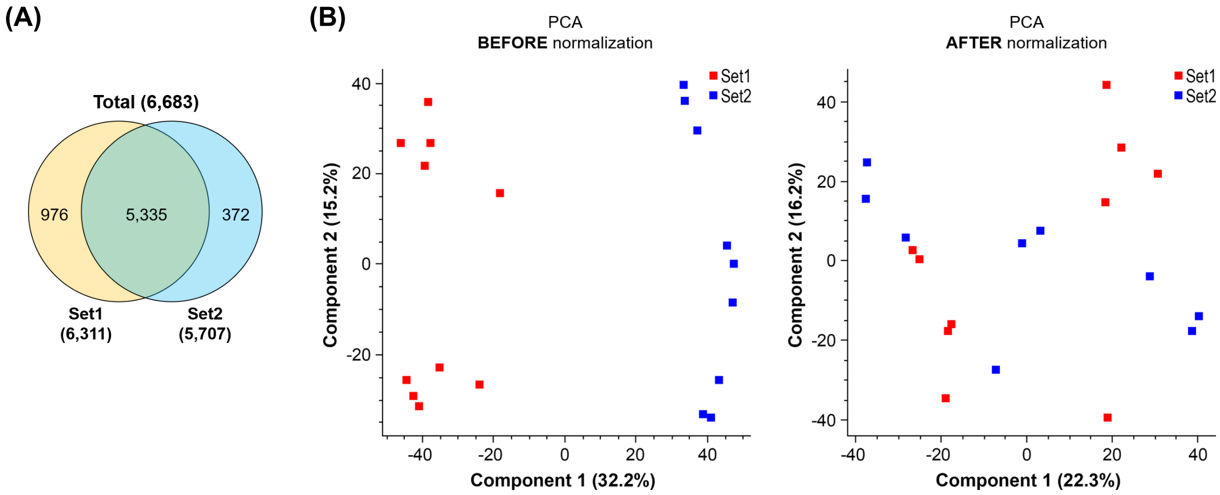

Supplemental Figure S6

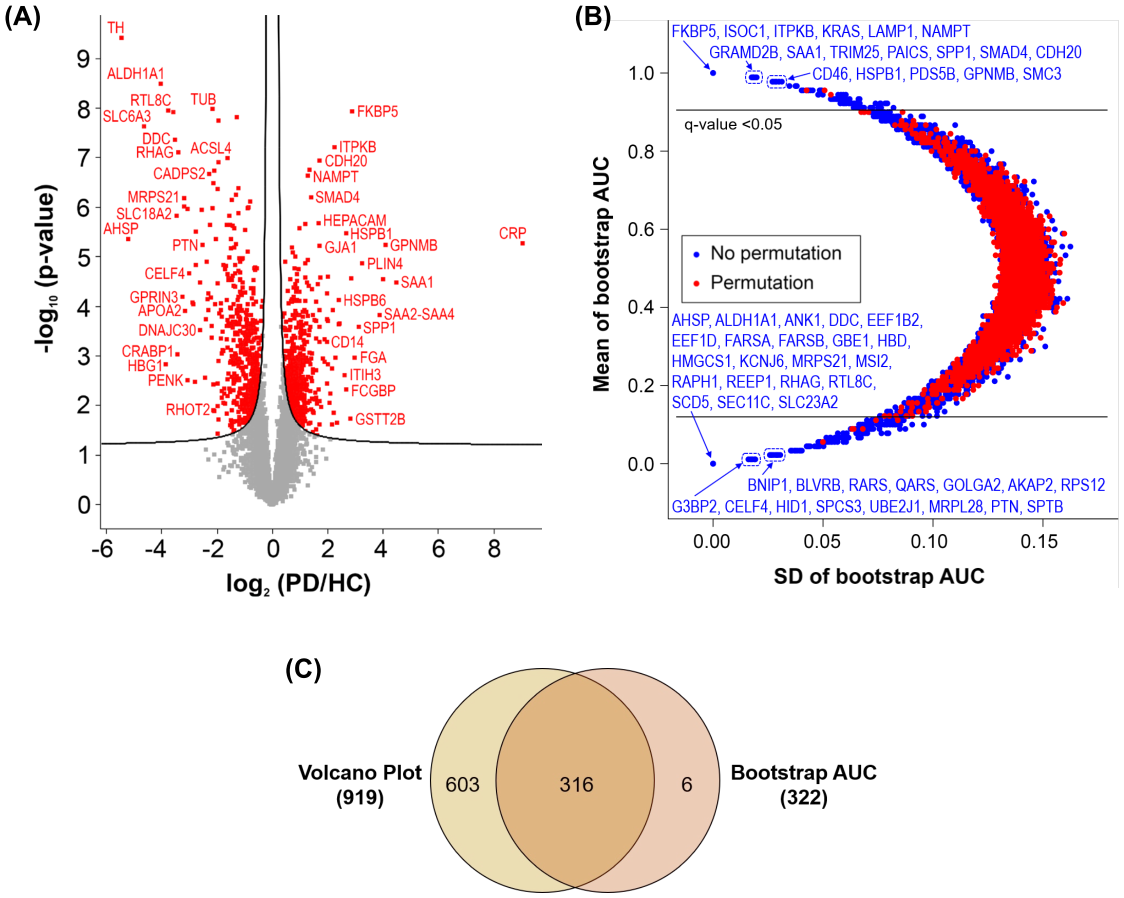

Supplemental Figure S7

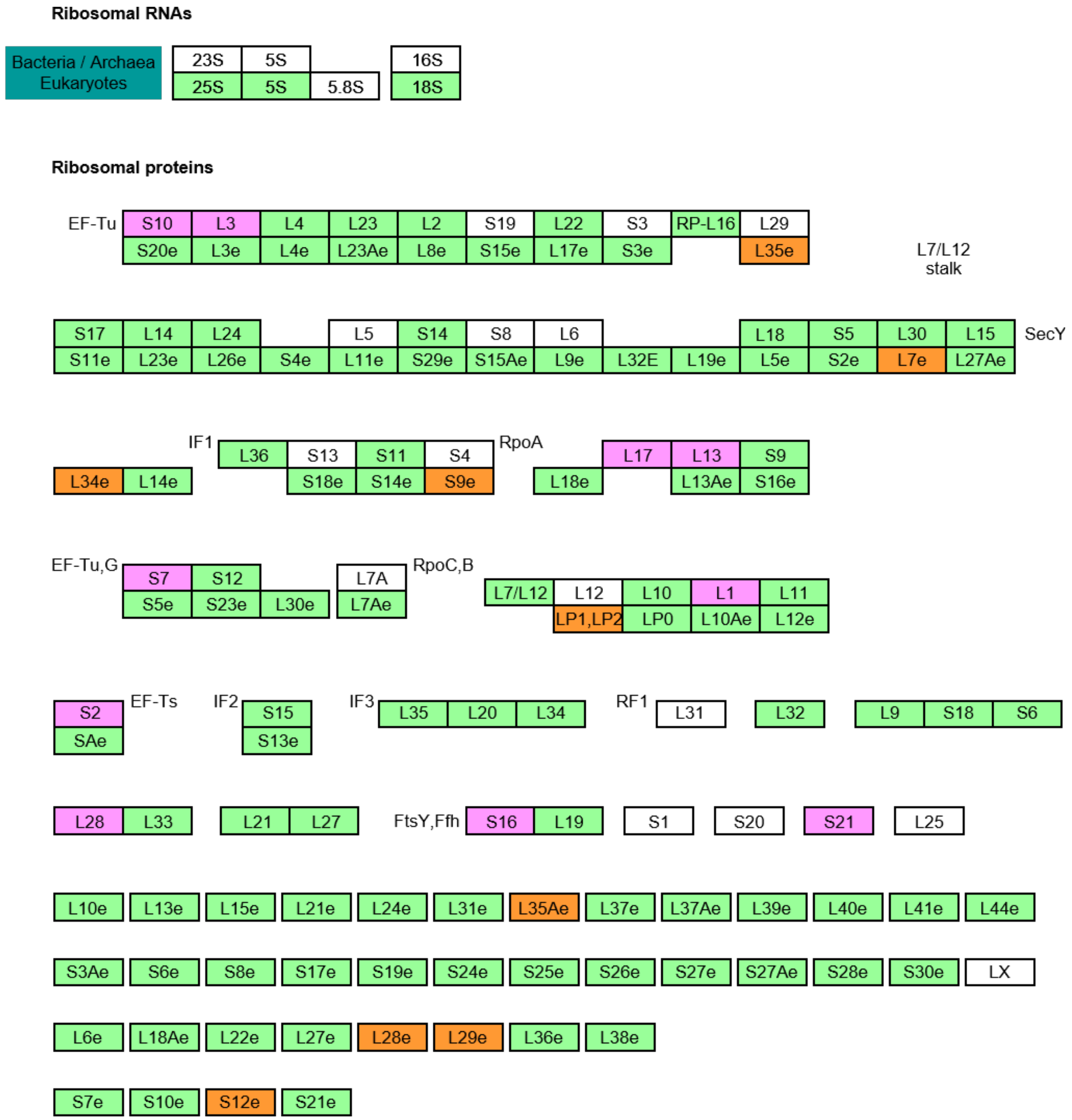

Supplemental Figure S8

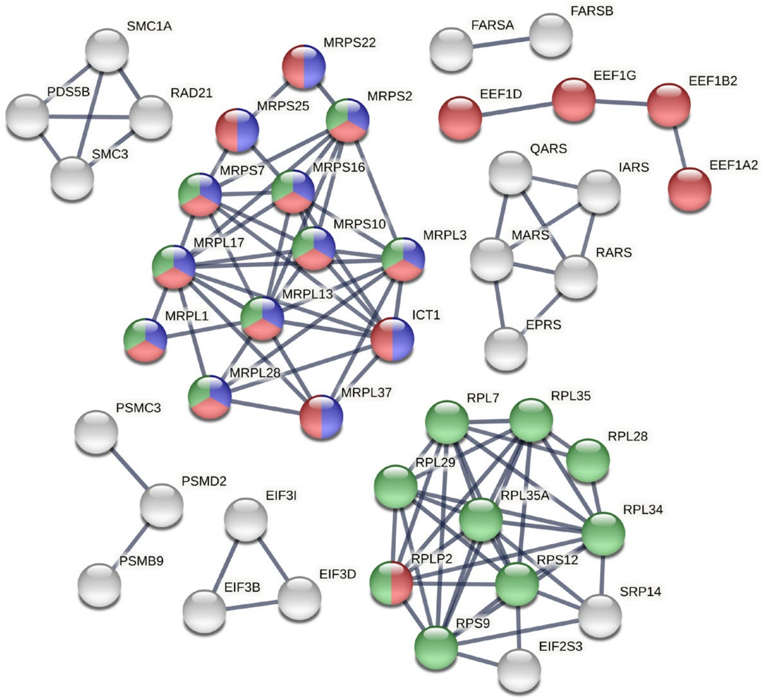

Supplement: Supplemental Figures S1–S8 [file mmc8.pdf]
