# Supplementary material for: Mass Spectrometry–Based Proteomics Analysis of Human Substantia Nigra From Parkinson's Disease Patients Identifies Multiple Pathways Potentially Involved in the Disease
Source: Mol Cell Proteomics. 2022 Nov 22;22(1):100452. doi: 10.1016/j.mcpro.2022.100452 (PMC9792365; doi:10.1016/j.mcpro.2022.100452)
Supplement: Supplemental Tables S1–S9 [file mmc9.docx]

**Supplemental Table S1. Information on the SN samples used in the replication study.**

| No. | Diagnosis | Age at death | Sex | Race | PMD (h) |
| --- | --- | --- | --- | --- | --- |
| 1 | PD with DEMENTIA | 65 | M | W | 21 |
| 2 | PD | 69 | M | W | - |
| 3 | PD | 72 | M | W | 15 |
| 4 | PD with DEMENTIA | 73 | M | W | 6.5 |
| 5 | PD with DEMENTIA, AD possible | 76 | M | W | 29 |
| 6 | LEWY BODY DISEASE, INCIPIENT AD | 86 | M | W | 19 |
| 7 | PD, NF DEGEN/TAU BRAAK 4, TBI POSSIBLE | 90 | M | W | 7 |
| 8 | PD, AD, MIXED DEMENTIA AD + PD | 91 | M | W | 19 |
| 9 | MIXED DEMENTIA (AD+PD), PD with DEMENTIA, AD PROB | 82 | M | W | - |
| 10 | PD with DEMENTIA | 63 | F | W | 14 |
| 11 | CONTROL, Tau MIN | 55 | M | W | 16 |
| 12 | CONTROL | 35 | M | W | 34 |
| 13 | CONTROL, TAU MIN | 46 | M | B | 24 |
| 14 | CONTROL, normal microscopic examination | 45 | F | W | 29 |
| 15 | CONTROL, TAU MIN | 55 | M | B | 32 |
| 16 | CONTROL, TAU MIN | 25 | F | W | 28 |
| 17 | CONTROL | 35 | M | W | 15 |
| 18 | CONTROL, TAU MIN | 33 | F | W | 17 |
| 19 | CONTROL, TAU MIN | 68 | F | W | 23 |

(M: male, F: female, W: white, B: black, PMD: postmortem delay, AD: Alzheimer disease, MIN: minimal, TBI: traumatic brain injury)

**Supplemental Table S2. Differentially expressed proteins in PD compared to HC calculated by SAM (top 50).**

| Protein name | Protein symbol | P value | *q*-value | z-score (PD/HC) |
| --- | --- | --- | --- | --- |
| Serum amyloid A-1 protein | SAA1 | 2.52E-07 | 0 | 8.42 |
| C-reactive protein | CRP | 2.14E-06 | 0 | 5.95 |
| Small EDRK-rich factor 2 | SERF2 | 2.29E-08 | 0 | 4.43 |
| Metallothionein-1F | MT1F | 2.44E-08 | 0 | 3.54 |
| Guanine nucleotide-binding protein G(I)/G(S)/G(O) subunit gamma-5 | GNG5 | 6.13E-07 | 0 | 3.07 |
| CD63 antigen | CD63 | 1.14E-08 | 0 | 2.98 |
| Prothymosin alpha (Fragment) | PTMA | 1.06E-06 | 0 | 2.91 |
| Metallothionein-2 | MT2A | 2.09E-06 | 0 | 2.67 |
| Perilipin-4 | PLIN4 | 8.59E-07 | 0 | 2.26 |
| Vesicle transport protein SFT2B | SFT2D2 | 3.01E-07 | 0 | 2.25 |
| Adenosylhomocysteinase (Fragment) | AHCYL2 | 4.69E-07 | 0 | 2.01 |
| NTF2-related export protein 1 | NXT1 | 2.22E-10 | 0 | 1.86 |
| Methylmalonyl-CoA epimerase, mitochondrial | MCEE | 3.30E-07 | 0 | 1.85 |
| D-aminoacyl-tRNA deacylase 1 | DTD1 | 2.92E-06 | 0 | 1.77 |
| Mothers against decapentaplegic homolog 4 | SMAD4 | 1.64E-06 | 0 | 1.76 |
| Syndecan-4 | SDC4 | 8.28E-08 | 0 | 1.74 |
| Calcium-binding and coiled-coil domain-containing protein 2 | CALCOCO2 | 3.09E-06 | 0 | 1.71 |
| MOB kinase activator 3B | MOB3B | 2.95E-06 | 0 | 1.70 |
| Ras-related protein Rab-8B | RAB8B | 2.74E-07 | 0 | 1.67 |
| Signal recognition particle 14 kDa protein | SRP14 | 1.11E-06 | 0 | 1.65 |
| Equilibrative nucleoside transporter 1 | SLC29A1 | 1.10E-06 | 0 | 1.63 |
| Putative RNA-binding protein Luc7-like 2 | LUC7L2 | 2.74E-09 | 0 | 1.60 |
| EMILIN-3 | EMILIN3 | 1.32E-07 | 0 | 1.57 |
| Caspase activity and apoptosis inhibitor 1 | CAAP1 | 1.51E-08 | 0 | 1.57 |
| Tropomyosin alpha-4 chain | TPM4 | 1.30E-06 | 0 | 1.49 |
| Histone H2AX | H2AFX | 3.17E-07 | 0 | 1.45 |
| Adenosylhomocysteinase 3 | AHCYL2 | 6.97E-07 | 0 | 1.38 |
| PEST proteolytic signal-containing nuclear protein | PCNP | 3.58E-08 | 0 | 1.30 |
| Tumor protein D54 | TPD52L2 | 4.82E-10 | 0 | 1.18 |
| 39S ribosomal protein L37, mitochondrial | MRPL37 | 2.60E-08 | 0 | -1.14 |
| Graves disease carrier protein | SLC25A16 | 3.76E-07 | 0 | -1.23 |
| 28S ribosomal protein S10, mitochondrial | MRPS10 | 6.81E-07 | 0 | -1.43 |
| Leucyl-cystinyl aminopeptidase | LNPEP | 4.84E-07 | 0 | -1.44 |
| UPF0577 protein KIAA1324-like | KIAA1324L | 3.95E-07 | 0 | -1.49 |
| 28S ribosomal protein S25, mitochondrial | MRPS25 | 1.09E-06 | 0 | -1.52 |
| 39S ribosomal protein L3, mitochondrial | MRPL3 | 4.22E-07 | 0 | -1.53 |
| 39S ribosomal protein L41, mitochondrial | MRPL41 | 2.26E-06 | 0 | -1.57 |
| 28S ribosomal protein S9, mitochondrial | MRPS9 | 7.78E-08 | 0 | -1.58 |
| 28S ribosomal protein S34, mitochondrial | MRPS34 | 2.61E-06 | 0 | -1.63 |
| 39S ribosomal protein L21, mitochondrial | MRPL21 | 2.46E-06 | 0 | -1.73 |
| 39S ribosomal protein L13, mitochondrial | MRPL13 | 1.91E-09 | 0 | -1.78 |
| HCG1984214, isoform CRA_a | hCG_1984214 | 8.20E-07 | 0 | -1.85 |
| Small G protein signaling modulator 3 | SGSM3 | 3.01E-06 | 0 | -1.98 |
| 39S ribosomal protein L28, mitochondrial | MRPL28 | 1.51E-09 | 0 | -2.24 |
| 28S ribosomal protein S24, mitochondrial | MRPS24 | 3.57E-08 | 0 | -2.24 |
| ELAV-like protein | ELAVL2 | 1.10E-07 | 0 | -2.25 |
| Copine-9 | CPNE9 | 8.53E-07 | 0 | -2.31 |
| 28S ribosomal protein S21, mitochondrial | MRPS21 | 2.51E-07 | 0 | -2.81 |
| Sodium-dependent dopamine transporter | SLC6A3 | 4.99E-07 | 0 | -3.09 |
| Retrotransposon Gag-like protein 8B | RTL8C | 1.77E-08 | 0 | -3.19 |

**Supplemental Table S3. Differentially expressed proteins in PD compared to HC calculated by Bootstrap AUC analysis (top 50).**

| Protein name | Protein symbol | P value | *q*-value | Mean^a^ | SD^b^ |
| --- | --- | --- | --- | --- | --- |
| Tumor protein D54 | TPD52L2 | 1.29E-08 | 0 | 1.000 | 0.0000 |
| Eukaryotic translation initiation factor 4B | EIF4B | 2.58E-08 | 0 | 0.996 | 0.0069 |
| CD63 antigen | CD63 | 2.58E-08 | 0 | 0.996 | 0.0075 |
| Methylmalonyl-CoA epimerase, mitochondrial | MCEE | 5.16E-08 | 0 | 0.991 | 0.0113 |
| Vesicle-associated membrane protein-associated protein A | VAPA | 9.03E-08 | 0 | 0.987 | 0.0158 |
| Putative RNA-binding protein Luc7-like 2 | LUC7L2 | 9.03E-08 | 0 | 0.987 | 0.0162 |
| PEST proteolytic signal-containing nuclear protein | PCNP | 2.45E-07 | 0 | 0.978 | 0.0189 |
| Metallothionein-1F | MT1F | 2.45E-07 | 0 | 0.978 | 0.0212 |
| Small EDRK-rich factor 2 | SERF2 | 2.45E-07 | 0 | 0.978 | 0.0236 |
| NTF2-related export protein 1 | NXT1 | 2.45E-07 | 0 | 0.978 | 0.0254 |
| Nipped-B-like protein | NIPBL | 3.87E-07 | 0 | 0.973 | 0.0215 |
| Syndecan-4 | SDC4 | 3.87E-07 | 0 | 0.973 | 0.0247 |
| A-kinase anchor protein 8-like | AKAP8L | 5.80E-07 | 0 | 0.969 | 0.0269 |
| Eukaryotic translation initiation factor 5B | EIF5B | 5.80E-07 | 0 | 0.969 | 0.0281 |
| Serum amyloid A-1 protein | SAA1 | 5.80E-07 | 0 | 0.969 | 0.0327 |
| Calcium-binding and coiled-coil domain-containing protein 2 | CALCOCO2 | 8.64E-07 | 0 | 0.964 | 0.0283 |
| Ras-related protein Rab-8B | RAB8B | 8.64E-07 | 0 | 0.964 | 0.0285 |
| Caspase activity and apoptosis inhibitor 1 | CAAP1 | 8.64E-07 | 0 | 0.964 | 0.0297 |
| EMILIN-3 | EMILIN3 | 8.64E-07 | 0 | 0.964 | 0.0336 |
| Adenosylhomocysteinase (Fragment) | AHCYL2 | 1.25E-06 | 0 | 0.960 | 0.0340 |
| Eukaryotic peptide chain release factor GTP-binding subunit ERF3A | GSPT1 | 1.25E-06 | 0 | 0.960 | 0.0342 |
| Equilibrative nucleoside transporter 1 | SLC29A1 | 1.79E-06 | 0 | 0.956 | 0.0319 |
| Mediator of RNA polymerase II transcription subunit 28 (Fragment) | MED28 | 1.79E-06 | 0 | 0.956 | 0.0324 |
| Translation initiation factor eIF-2B subunit epsilon | EIF2B5 | 1.79E-06 | 0 | 0.044 | 0.0335 |
| AKT-interacting protein | AKTIP | 1.79E-06 | 0 | 0.044 | 0.0337 |
| Cadherin-10 | CDH10 | 1.25E-06 | 0 | 0.040 | 0.0311 |
| Polymerase delta-interacting protein 2 | POLDIP2 | 1.25E-06 | 0 | 0.040 | 0.0331 |
| ELAV-like protein | ELAVL2 | 1.25E-06 | 0 | 0.040 | 0.0342 |
| Transmembrane protein 33 | TMEM33 | 8.64E-07 | 0 | 0.036 | 0.0285 |
| UPF0577 protein KIAA1324-like | KIAA1324L | 8.64E-07 | 0 | 0.036 | 0.0287 |
| Graves disease carrier protein | SLC25A16 | 8.64E-07 | 0 | 0.036 | 0.0288 |
| Glycosylphosphatidylinositol anchor attachment 1 protein | GPAA1 | 8.64E-07 | 0 | 0.036 | 0.0292 |
| Receptor tyrosine-protein kinase erbB-4 | ERBB4 | 8.64E-07 | 0 | 0.036 | 0.0304 |
| Long-chain-fatty-acid--CoA ligase 4 | ACSL4 | 5.80E-07 | 0 | 0.031 | 0.0262 |
| Golgi to ER traffic protein 4 homolog | GET4 | 5.80E-07 | 0 | 0.031 | 0.0281 |
| 39S ribosomal protein L38, mitochondrial | MRPL38 | 3.87E-07 | 0 | 0.027 | 0.0226 |
| 39S ribosomal protein L16, mitochondrial | MRPL16 | 3.87E-07 | 0 | 0.027 | 0.0239 |
| Palmitoyltransferase ZDHHC17 | ZDHHC17 | 3.87E-07 | 0 | 0.027 | 0.0240 |
| Copine-9 | CPNE9 | 3.87E-07 | 0 | 0.027 | 0.0258 |
| ELAV-like protein | ELAVL4 | 3.87E-07 | 0 | 0.027 | 0.0267 |
| 28S ribosomal protein S24, mitochondrial | MRPS24 | 2.45E-07 | 0 | 0.022 | 0.0213 |
| Putative mitochondrial import inner membrane translocase subunit Tim23B | TIMM23B | 2.45E-07 | 0 | 0.022 | 0.0219 |
| 39S ribosomal protein L3, mitochondrial | MRPL3 | 2.45E-07 | 0 | 0.022 | 0.0220 |
| Leucyl-cystinyl aminopeptidase | LNPEP | 2.45E-07 | 0 | 0.022 | 0.0231 |
| 39S ribosomal protein L13, mitochondrial | MRPL13 | 2.45E-07 | 0 | 0.022 | 0.0232 |
| 39S ribosomal protein L37, mitochondrial | MRPL37 | 9.03E-08 | 0 | 0.013 | 0.0149 |
| 28S ribosomal protein S9, mitochondrial | MRPS9 | 9.03E-08 | 0 | 0.013 | 0.0160 |
| Retrotransposon Gag-like protein 8B | RTL8C | 9.03E-08 | 0 | 0.013 | 0.0162 |
| HCG1984214, isoform CRA_a | hCG_1984214 | 5.16E-08 | 0 | 0.009 | 0.0124 |
| 39S ribosomal protein L28, mitochondrial | MRPL28 | 2.58E-08 | 0 | 0.004 | 0.0074 |

^a^ Mean represents the means of AUCs from the bootstrap ROC analysis. The proteins with the Mean values > 0.5 and < 0.5 are up-regulated and down-regulated in PD, respectively.

^b^ SD represents the standard deviations of AUCs from the bootstrap ROC analysis.

**Supplemental Table S4. List of proteins in each enriched pathway.**

| Pathways | Protein symbol |
| --- | --- |
| Ribosome (42) | MRPL1, MRPL10, MRPL11, MRPL13, MRPL14, MRPL16, MRPL17, MRPL19, MRPL20, MRPL21, MRPL22, MRPL23, MRPL24, MRPL27, MRPL28, MRPL3, MRPL30, MRPL4, MRPS10, MRPS15, MRPS16, MRPS21, MRPS6, MRPS7, MRPS9, RPL10A, RPL11, RPL30, RPL35, RPL36AL, RPL37A, RPL38, RPL9, RPLP0, RPLP1, RPLP2, RPS12, RPS14, RPS20, RPS27L, RPS28, RPSA |
| GABAergic synapse (18) | ADCY3, GABARAP, GABBR1, GABBR2, GABRA1, GABRA2, GABRA3, GABRA4, GABRB1, GLS, GNG10, GNG3, GNG5, KCNJ6, PRKACA, PRKACB, PRKCA, SLC38A3 |
| Retrograde endocannabinoid signaling (18) | ADCY3, GABRA1, GABRA2, GABRA3, GABRA4, GABRB1, GNAQ, GNG10, GNG3, GNG5, GRIA1, KCNJ6, MAPK10, MAPK9, PLCB2, PRKACA, PRKACB, PRKCA |
| Cell adhesion molecules (CAMs) (22) | CD276, CD58, CDH2, CLDN11, CLDN5, ESAM, F11R, ITGAV, ITGB1, ITGB8, JAM3, MPZ, MPZL1, NCAM1, NECTIN2, NEO1, NLGN1, PECAM1, PTPRC, PVR, SDC1, SDC4 |
| Morphine addiction (16) | ADCY3, GABBR1, GABBR2, GABRA1, GABRA2, GABRA3, GABRA4, GABRB1, GNG10, GNG3, GNG5, GRK2, KCNJ6, PRKACA, PRKACB, PRKCA |
| Prion diseases (9) | BAX, C1QA, C1QB, C1QC, C9, NCAM1, PRKACA, PRKACB, STIP1 |
| Parkinson's disease (20) | COX6B1, COX7B, GPR37, NDUFA1, NDUFA4L2, NDUFAB1, NDUFB2, NDUFB3, NDUFB9, NDUFC1, PPIF, PRKACA, PRKACB, SLC18A2, SLC6A3, TH, UBE2L3, UQCRH, UQCRQ, VDAC3 |

**Supplemental Table S5. List of ribosomal proteins among the differentially expressed proteins in PD.**

| Protein name | Protein symbol | -log10 (P value) | z-score (PD/HC) |
| --- | --- | --- | --- |
| 60S ribosomal protein L36a-like | RPL36AL | 5.299418 | 2.42 |
| 60S ribosomal protein L37a | RPL37A | 2.195736 | 1.75 |
| 60S ribosomal protein L30 | RPL30 | 2.531979 | -0.46 |
| 60S ribosomal protein L11 | RPL11 | 3.716187 | -0.61 |
| 60S ribosomal protein L38 | RPL38 | 3.79038 | -0.65 |
| 60S acidic ribosomal protein P0 | RPLP0 | 3.906003 | -0.72 |
| 40S ribosomal protein S27-like | RPS27L | 2.682879 | -0.76 |
| 40S ribosomal protein S20 | RPS20 | 3.056974 | -0.76 |
| 60S acidic ribosomal protein P2 | RPLP2 | 4.58672 | -0.78 |
| 40S ribosomal protein S28 | RPS28 | 4.653765 | -0.82 |
| 40S ribosomal protein S14 | RPS14 | 3.409694 | -0.83 |
| 60S ribosomal protein L9 | RPL9 | 5.63534 | -0.84 |
| 40S ribosomal protein SA | RPSA | 5.794589 | -0.86 |
| 40S ribosomal protein S12 | RPS12 | 5.329034 | -0.91 |
| 60S ribosomal protein L10a | RPL10A | 5.777511 | -1.00 |
| 60S acidic ribosomal protein P1 | RPLP1 | 5.952305 | -1.00 |
| 60S ribosomal protein L35 | RPL35 | 2.717001 | -1.24 |

**Supplemental Table S6. List of mitochondrial ribosomal proteins among the differentially expressed proteins in PD.**

| Protein names | Protein symbol | -log10 (P value) | z-score (PD/HC) |
| --- | --- | --- | --- |
| 28S ribosomal protein S21, mitochondrial | MRPS21 | 6.60101 | -2.81 |
| 39S ribosomal protein L28, mitochondrial | MRPL28 | 8.82215 | -2.24 |
| 39S ribosomal protein L23, mitochondrial | MRPL23 | 4.29004 | -1.81 |
| 39S ribosomal protein L13, mitochondrial | MRPL13 | 8.71845 | -1.78 |
| 39S ribosomal protein L21, mitochondrial | MRPL21 | 5.60843 | -1.73 |
| 28S ribosomal protein S9, mitochondrial | MRPS9 | 7.1089 | -1.58 |
| 28S ribosomal protein S16, mitochondrial | MRPS16 | 4.8268 | -1.55 |
| 28S ribosomal protein S7, mitochondrial | MRPS7 | 5.50371 | -1.55 |
| 39S ribosomal protein L3, mitochondrial | MRPL3 | 6.3742 | -1.53 |
| 39S ribosomal protein L24, mitochondrial | MRPL24 | 5.00244 | -1.47 |
| 28S ribosomal protein S10, mitochondrial | MRPS10 | 6.16701 | -1.43 |
| 39S ribosomal protein L19, mitochondrial | MRPL19 | 5.24302 | -1.15 |
| 39S ribosomal protein L1, mitochondrial | MRPL1 | 3.88352 | -1.04 |
| 28S ribosomal protein S15, mitochondrial | MRPS15 | 3.49333 | -1.00 |
| 39S ribosomal protein L16, mitochondrial | MRPL16 | 6.68681 | -0.98 |
| 28S ribosomal protein S6, mitochondrial | MRPS6 | 3.74684 | -0.97 |
| 39S ribosomal protein L30, mitochondrial | MRPL30 | 3.22197 | -0.95 |
| 39S ribosomal protein L20, mitochondrial | MRPL20 | 4.63811 | -0.90 |
| 39S ribosomal protein L10, mitochondrial | MRPL10 | 3.92776 | -0.90 |
| 39S ribosomal protein L17, mitochondrial | MRPL17 | 4.99382 | -0.84 |
| 39S ribosomal protein L27, mitochondrial | MRPL27 | 3.51562 | -0.81 |
| 39S ribosomal protein L11, mitochondrial | MRPL11 | 3.06887 | -0.79 |
| 39S ribosomal protein L22, mitochondrial | MRPL22 | 5.1938 | -0.77 |
| 39S ribosomal protein L14, mitochondrial | MRPL14 | 2.62518 | -0.75 |
| 39S ribosomal protein L4, mitochondrial | MRPL4 | 2.40357 | -0.55 |

**Supplemental Table S7. List of enriched pathways for the protein in Blue (M21) and Salmon (M23) modules generated by WGCNA.**

| Term (Blue: M21) | Count/PH | % | P value |
| --- | --- | --- | --- |
| Ribosome | 45/136 | 33.1 | 3.20E-20 |
| Protein processing in endoplasmic reticulum | 31/169 | 18.3 | 3.50E-07 |
| Aminoacyl-tRNA biosynthesis | 16/66 | 24.2 | 1.60E-05 |
| Proteasome | 11/44 | 25.0 | 4.10E-04 |
| Nicotine addiction | 10/40 | 25.0 | 8.70E-04 |

| Term (Salmon: M23) | Count/PH | % | P value |
| --- | --- | --- | --- |
| Ribosome | 22/136 | 16.2 | 5.80E-17 |
| Adherens junction | 6/71 | 8.5 | 2.90E-03 |
| Rap1 signaling pathway | 8/210 | 3.8 | 2.60E-02 |
| RNA transport | 7/172 | 4.1 | 3.10E-02 |
| Non-alcoholic fatty liver disease (NAFLD) | 6/151 | 4.0 | 5.70E-02 |

(PH: the total number of proteins in the pathway)

**Supplemental Table S8. List of enriched pathways for the differentially expressed proteins in PD compared to HC of the replication experiment data set.**

| Term | Count/PH | % | P value |
| --- | --- | --- | --- |
| Ribosome | 19/136 | 14.0 | 6.80E-10 |
| Aminoacyl-tRNA biosynthesis | 9/66 | 13.6 | 8.70E-05 |
| Dopaminergic synapse | 9/128 | 7.0 | 6.90E-03 |
| Protein export | 4/23 | 17.4 | 1.30E-02 |
| Amphetamine addiction | 6/66 | 9.1 | 1.50E-02 |
| PPAR signaling pathway | 6/67 | 9.0 | 1.50E-02 |
| Cocaine addiction | 5/49 | 10.2 | 2.20E-02 |

(PH: the total number of proteins in the pathway)

**Supplemental Table S9. List of glutamate receptor proteins identified in this study.**

|  | SAM analysis | | | Bootstrap AUC analysis | | |
| --- | --- | --- | --- | --- | --- | --- |
| Protein symbol | P value | *q*-value | z-score (PD/HC) | Mean of bootstrap AUC | SD of bootstrap AUC | *q*-value |
| GRIA1 | 0.000385 | 0.014549 | -0.57097 | 0.151111 | 0.068768 | 0 |
| GRIN2B | 0.005691 | 0.027773 | -0.74256 | 0.222222 | 0.088551 | 0.061709 |
| GRIA2 | 0.006059 | 0.035713 | -0.62962 | 0.235556 | 0.090238 | 0.074803 |
| GRIK3 | 0.020968 | 0.094342 | -0.46308 | 0.24 | 0.091827 | 0.083228 |
| GRIA3 | 0.017949 | 0.097337 | -0.42797 | 0.248889 | 0.090441 | 0.091629 |
| GRIN1 | 0.061383 | 0.134924 | -0.53172 | 0.32 | 0.095923 | 0.265215 |
| GRM3 | 0.11086 | 0.145862 | -0.89208 | 0.351111 | 0.108161 | 0.367172 |
| GRIN2A | 0.057711 | 0.184823 | -0.35383 | 0.302222 | 0.097546 | 0.201916 |
| GRIN2D | 0.078849 | 0.194964 | -0.39044 | 0.337778 | 0.102652 | 0.318283 |
| GRID1 | 0.036518 | 0.232384 | -0.24035 | 0.28 | 0.09466 | 0.148918 |
| GRIP1 | 0.057174 | 0.263294 | -0.23538 | 0.288889 | 0.095103 | 0.170161 |
| GRM8 | 0.262679 | 0.367606 | -0.38496 | 0.368889 | 0.103856 | 0.435933 |
| GRID2 | 0.279154 | 0.442437 | -0.24287 | 0.373333 | 0.103688 | 0.462264 |
| GRM4 | 0.436785 | 0.543541 | -0.28244 | 0.422222 | 0.106323 | 0.69773 |
| GRIA4 | 0.420806 | 0.592332 | 0.166854 | 0.591111 | 0.109189 | 0.586477 |
| GRM5 | 0.61185 | 0.730387 | 0.127857 | 0.484444 | 0.112226 | 0.96377 |
| GRM1 | 0.739105 | 0.804884 | -0.14692 | 0.502222 | 0.104994 | 0.969804 |
| GRIK2 | 0.778885 | 0.865328 | 0.061072 | 0.506667 | 0.116312 | 0.960577 |
| GRM2 | 0.841769 | 0.893245 | -0.06589 | 0.484444 | 0.110263 | 0.973998 |
| GRM7 | 0.875562 | 0.917452 | -0.05058 | 0.457778 | 0.10707 | 0.863437 |
